# Unravelling the Photoprotective Mechanisms of Nature-Inspired Ultraviolet Filters Using Ultrafast Spectroscopy

**DOI:** 10.3390/molecules25173945

**Published:** 2020-08-28

**Authors:** Temitope T. Abiola, Abigail L. Whittock, Vasilios G. Stavros

**Affiliations:** 1 Department of Chemistry, University of Warwick, Coventry CV4 7AL, UK; temitope.abiola@warwick.ac.uk (T.T.A.); abbie.whittock@warwick.ac.uk (A.L.W.); 2 AS CDT, Senate House, University of Warwick, Coventry CV4 7AL, UK

**Keywords:** ultrafast spectroscopy, sunscreens, nature-inspired, photoprotection, photochemistry, photophysics

## Abstract

There are several drawbacks with the current commercially available ultraviolet (UV) filters used in sunscreen formulations, namely deleterious human and ecotoxic effects. As a result of the drawbacks, a current research interest is in identifying and designing new UV filters. One approach that has been explored in recent years is to use nature as inspiration, which is the focus of this review. Both plants and microorganisms have adapted to synthesize their own photoprotective molecules to guard their DNA from potentially harmful UV radiation. The relaxation mechanism of a molecule after it has been photoexcited can be unravelled by several techniques, the ones of most interest for this review being ultrafast spectroscopy and computational methods. Within the literature, both techniques have been implemented on plant-, and microbial-inspired UV filters to better understand their photoprotective roles in nature. This review aims to explore these findings for both families of nature-inspired UV filters in the hope of guiding the future design of sunscreens.

## 1. Introduction

### 1.1. Ultraviolet Radiation and Biological Systems

Ultraviolet radiation (UVR; 400–100 nm) is the most energetic region of the broad spectrum of wavelengths that reach the Earth from solar radiation. This radiation is further subdivided into ultraviolet (UV)-A (400–315 nm), UV-B (315–280 nm) and UV-C (280–100 nm) [1,2]. Almost all the UV-C and a large fraction of the UV-B radiation are absorbed by the ozone layer in the stratosphere. This results in 5% UV-B and 95% UV-A accounting for the total UVR that reaches the Earth’s surface. These energy components impact on the Earth’s biosphere [3,4]. Previous physiological response studies in humans revealed that there are vital benefits to UVR exposure—the production of vitamin D, which is essential for prevention of osteoporosis and skeletal disease [5,6,7], and improving symptoms of mental health conditions such as seasonal affective disorder and schizophrenia [8,9]. However, the damaging effects of overexposure to UVR have been widely reported in previous studies and reviews—these include cataract formation, skin ageing, DNA mutation, and skin cancer [10,11,12,13,14,15,16,17]. Hence, the need for a balance between exposure to UVR and protection against overexposure to UV-A and UV-B is of crucial importance.

The human body possesses many natural defence mechanisms to reduce the effects of UVR exposure, such as skin pigmentation, made up of a class of UV-absorbing molecules, termed melanin. Although melanin absorbs UVR before it reaches vulnerable DNA in the skin, it offers insufficient protection for the skin on exposure to high levels of UVR [11,18,19]. Furthermore, the production of extra melanin triggered by exposure to UVR (i.e., tanning) is a delayed process and may take 3–5 days to provide any significant photoprotection [20]. Therefore, there is a need for commercial UV filters that can provide an immediate form of photoprotection on the skin.

#### 1.1.1. Previous Attempt to Address Sun Protection

Human efforts to address photoprotection date back to the Ancient Egyptians and Greeks, who sought to protect themselves from sunburn [11]. Although the deleterious effects of solar radiation on the skin were not well characterized at the time, the concept of tanning was well understood. In an attempt to keep their skin lighter, olive oil, jasmine, lupine and rice bran were used on the skin as cosmetics. Reports suggest that rice bran absorbs UVR, jasmine helps in the repair of DNA, while lupine lightens the skin [11,21,22,23]. However, it was the identification of UVR by Ritter in 1801 and the experimental works of Widmark in 1889, proving that erythema solare (sunburn) is caused by UVR, which gave rise to the interest in finding UV filters to protect the skin from photodamage [11,24]. By the end of the 19^th^ century, the suggestion to design and use chemical-based UV filters to prevent photodamage was already gaining attention [11,24,25]. Schueller in 1935 formulated one of the first commercial sunscreens called “Ambre Solaire,” containing the UV filter benzyl salicylate. Thereafter, many other potential sunscreen compounds were identified [24,25]. In recent years, both nature-inspired and artificial UV filters have been studied in an attempt to address photoprotection. 

Current commercial UV active ingredients in sunscreen formulations are broadly classified into two major groups: physical blockers (inorganic) and chemical absorbers (organic). Physical blockers such as titanium dioxide (TiO_2_) and zinc oxide (ZnO) block UV-A/UV-B radiation primarily through scattering. Chemical absorbers such as oxybenzone, avobenzone, homosalate, octocrylene and many others absorb UV-A/UV-B radiation before it reaches the skin [12,26]. 

#### 1.1.2. Challenges with Existing Sunscreens

Despite the many advantages of the vast number of commercial sunscreens available for photoprotection, continued research is still required to overcome the associated drawbacks with these products. These drawbacks include photoinstability, the limited number of approved UV-A filters, and environmental and dermatological effects to name but a few [27,28,29].

Protection against the damaging effects of UVR exposure requires that sunscreens be photostable (i.e., do not degrade after absorption of UVR). The publication by Kockler et al. [30] revealed that following exposure to the sun and irradiation with a UV lamp, several commercial sunscreens are photounstable in the UV-A region. A similar study by Gonzalez et al. [31] reported that several commercially available broadband sunscreen products are photounstable. The design of photostable sunscreen formulation remains a challenge in industry and for researchers to solve to attain an effective product for ultimate photoprotection.

The development of UV-B filters has received considerable attention over the years due to the higher energy of UV-B radiation compared to UV-A. Despite being less energetic, UV-A is very abundant at the Earth’s surface and penetrates much deeper into the skin than UV-B radiation, reaching far into the dermis [32,33]. The effects of UV-A radiation on humans have been reported as ranging from suppression of acquired immunity, DNA mutation and the production of reactive oxygen species which often facilitate skin ageing and carcinogenesis [34,35]. Since the effects of UV-A radiation on the skin have been reported, the sunscreen industry and regulatory agencies now desire a broadband sunscreen (i.e., sunscreens that span across both UV-B and UV-A). However, there are still only a few Food and Drug Administration (FDA)/European Union (EU)-approved UV-A filters and the most widely used (avobenzone) is not very photostable [34,35,36,37]. This implies that research is still required for the identification of effective UV-A filters.

The effect of sunscreens on environmental health have raised concern over the years, with environmentalists calling for the ban of some chemical UV filters. Several studies have described the developmental and reproductive toxicity of some widely used organic UV filters (oxybenzone, avobenzone and octocrylene) in sunscreen formulation to fish and corals [28,29,38,39]. Accumulation of organic UV filters was also found in soil, sediments [40] and aquatic biota such as clams, urchins, dolphins and fish [41,42]. In order to maintain a healthy ecosystem as well as human photoprotection against UVR, there is a need to develop sunscreens that are safe for the environment.

In a study designed to evaluate the allergens associated with sunscreen products, the North American Contact Dermatitis Group found and reported allergens from oxybenzone [43]. A similar report on oxybenzone and other sunscreen active ingredients was published in an opinion paper by the European Scientific Committee on Consumer Safety based on reviews of 20 publications on the dermatological effects of sunscreen agents [44]. Following the research reported by Heurung et al. [45], the American Contact Dermatitis Society in 2014 listed benzophenones as the allergen of the year, with oxybenzone showing the worst allergenic effects of the class. Matta et al. [46,47] studied the absorption and pharmacokinetics of commonly used organic UV filters in sunscreen formulations, such as avobenzone, oxybenzone, octocrylene, ecamsule, homosalate, octisalate and octinoxate, and these UV filters were reported to have achieved plasma concentrations significantly above the FDA threshold. Altogether, these allergen studies do not provide any evidence that organic UV filters are harmful. However, it raises some questions about their safety. Considering these drawbacks, research has been targeted towards designing photostable and safe UV filters for both humans and the environment. One way to achieve this is by using nature as inspiration for UV filter design.

#### 1.1.3. Nature-Inspired Sunscreens

In recent years, a popular research approach towards designing new UV filters has been nature-inspired, with a particular focus on plant- and microbial-inspired UV filters. As a result, the focus of this review is to explore the findings and potential use of nature-inspired UV filters towards the development of more efficient and safer sunscreens.

#### 1.1.4. Plant Ultraviolet Filters

Plant species have been reported to share a similar burden of disease from UVR as found in humans. Plants require sunlight for photosynthesis, hence the need for some UVR exposure; however, too much UVR can result in damaging effects [48]. Like humans, moderate exposure of plants to UVR has important implications; UV-B radiation in particular acts as a signal transducer for a vast array of processes that initiate or regulate gene responses in plants that are essential for survival [49]. Furthermore, UV-B radiation has been reported to stimulate the expression of genes responsible for UV protection and DNA repair, and therefore actively promotes survival in sunlight [49,50]. However, overexposure to UVR can result in a number of damaging effects, such as inhibition of growth, disruption of transpiration and photosynthesis. Other effects include damage of DNA, either by direct photodamage or indirectly through the generation of reactive oxygen species, which can, in turn, interact with DNA nucleotides [49,50,51]. Other reported damages include a reduction in the pollen fertility of some plants and photomorphogenesis of plant leaves, e.g., thickening of epidermis which increase susceptibility to invading pathogens [49,50]. On the other hand, underexposure to UVR (UV-B) could result in susceptibility of the plant to pathogens and a reduction in the UV-B signal transduction pathway that supports many photophysical processes supra [49,50].

**Figure 1:**
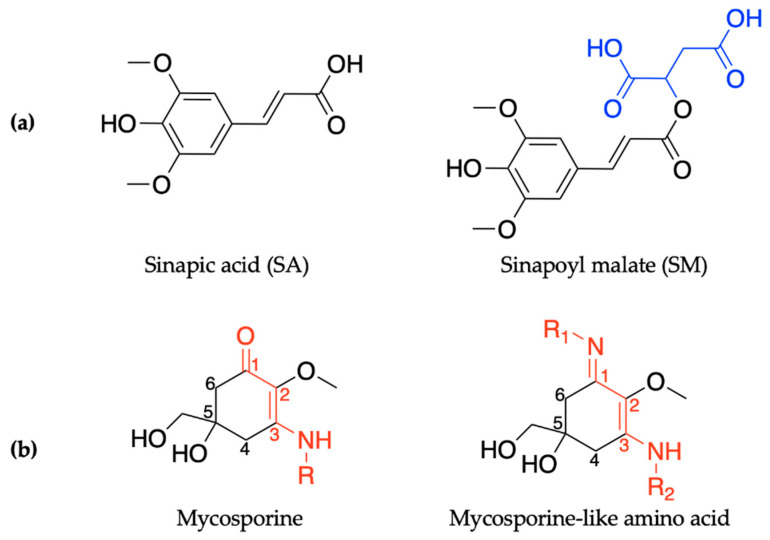
Chemical structures of ultraviolet (UV) filters found in nature including (**a**) the plant-based sinapic acid (SA) and sinapoyl malate (SM), with the malate group added to the sinapic acid shown in blue; and (**b**) the core components of mycosporines and mycosporine-like amino acids synthesized by microorganisms, with the chromophores shown in red. See main text for definitions of R/R_1_/R_2_. Stereocenters have not been included in the structures.

In order to maintain a balance in the extent of UVR exposure, plants have a regulated biochemical pathway termed the phenylpropanoid pathway which is similar to melanogenesis in humans [52]. The phenylpropanoid pathway is responsible for the synthesis of several metabolites used in numerous biochemical processes in plants. With respect to photoprotection, previous studies have shown that certain phenolic substances deposited in the vacuoles of cells in the upper epidermis of leaves, such as sinapoyl malate (SM) and the building block, sinapic acid (SA) (see Figure 1a), are the most prominent metabolites [12,53]. These molecules (SM and SA) act as UV-B filters by absorbing UVR and subsequently dissipating the UV energy (as heat) before it reaches sensitive cells in the leaves. For example, in thale cress (*Arabidopsis thaliana*), SM has been reported as the UV filter that reduces the potential adverse effect of excess UV-B radiation [12,52]. While early works on plant UV filters and their derivatives have been reviewed in detail in previous publications [54,55], the photochemistry and photophysics of the recently reported literature for these classes of UV filters will be covered in Section 2.1 of this review.

#### 1.1.5. Microbial Ultraviolet Filters

Similarly to plants and humans, microorganisms also need to protect themselves from harmful DNA damage that is caused by UVR [56,57]. Microorganisms such as cyanobacteria, fungi and micro- and macroalgae combat this problem by synthesising a family of secondary metabolites called mycosporines and mycosporine-like amino acids [58,59]. Structurally, mycosporines are composed of a cyclohexenone unit with different amino compounds bound at carbon three (relative to the carbonyl), represented as R in Figure 1b. Mycosporine-like amino acids are composed of a cyclohexenimine unit with an imino moiety at carbon one and an amino compound at carbon three, represented as R_1_ and R_2_, respectively, in Figure 1b [60]. Fungi only synthesize mycosporines, while marine microorganisms can synthesize both mycosporines and mycosporine-like amino acids [58]. Within the literature, mycosporines and mycosporine-like amino acids have been used synonymously, so both classes of molecules will be referred to as MAAs for the purpose of this introduction [60]. It is added here that the two classes of molecules are referred to separately again in Section 2.2 of this review.

The role of MAAs in biological processes is often debated but they are thought to span a broad range, from osmotic regulation, defence against oxidative stress, protection against thermal stress to serving as intracellular nitrogen reservoirs [56,61]. Furthermore, MAAs provide photoprotection against harmful UVR to the organisms that synthesize them [56,60,62,63,64]. The photoprotective properties of MAAs have been deduced from their efficient absorption in the UV region of the electromagnetic spectrum and because there is a correlation between MAA concentration and exposure to UVR [65,66]. There are more than 30 known MAAs to date [63], each with an absorption maximum in the UV-A or UV-B region (310–362 nm) and molar extinction coefficients in the range of 28,100 to 50,000 M^−1^ cm^−1^ [67,68], cf. 34 140 M^−1^ cm^−1^ for avobenzone [69]. As a result, MAAs are considered to be the strongest UV-A absorbers found in nature. In addition to their strong UV absorption, some MAAs, such as mycosporine-glycine, exhibit antioxidant properties that make them highly desirable to the commercial industry [61,62,70,71]. A recent publication by Rosic [68] reviewed some of the aforementioned roles of MAAs, and linked them to how MAAs could be used in the commercial industry to overcome some of the drawbacks with current UV filters covered in Section 1.1.2. It is noted here that several publications have identified the instability of some MAAs, in particular those with a cyclohexenone core, as they undergo hydrolysis of the amine on carbon three [72,73,74]. Whilst this is not the focus of the present review, it is an important factor that must be considered when determining a good UV filter, and is an avenue that certainly warrants future work.

There have been a number of studies on the photochemistry and photophysics of MAAs and these will be explored in some detail in this review [66,75,76,77,78,79,80,81,82] (for more detailed reviews on the photochemistry and photophysics of MAAs, the reader is directed to reviews by Losantos et al. [62] and Woolley and Stavros [83]). To date, there has been no published ultrafast spectroscopy measurements on natural MAAs. However, Losantos et al. [84] and Woolley et al. [85] have conducted such experiments on MAA motifs. In addition to these studies, complementary theoretical work has modelled the minimum energy relaxation pathways of MAAs and molecules alike, giving insight into their photoprotective mechanisms [84,86,87,88,89,90]. Section 2.2 of this review will primarily cover ultrafast spectroscopy and theoretical work conducted to the present date on MAAs and MAA motifs. In exploring such studies, the aim is to unravel the photoprotective mechanisms by which MAAs dissipate absorbed UVR.

### 1.2. Experimental Technique

#### 1.2.1. Femtosecond Pump–Probe Spectroscopy

Before the historic application of laser femtochemistry to monitor bond-breaking reactions by Zewail and co-workers [91], several approaches have been used by many research laboratories in an attempt to understand the dynamics of reactions (bond breaking and formation) [91]. These include the use of absorption, emission, scattering and ion spectroscopy to examine reaction coordinates. Compared to the time required for bond breaking and formation (picoseconds or less), these preliminary approaches could only record data on a longer timescale, thereby providing evidence about the initial and final processes occurring along the reaction coordinate without direct time resolution [91,92]. Improving on these approaches, Zewail and co-workers employed femtosecond time-resolution experiments to monitor the transition state of a photochemical reaction directly, using the ultrafast pump–probe technique. In this technique, the laser pump pulse is used to excite the molecule of interest to the excited state, from where relaxation back to the ground state can occur on the timescale of femtoseconds or picoseconds. After the pump pulse excites the sample of interest, a second probe laser pulse is used to track the excited-state population at various delay times relative to the initial pump pulse excitation [92]. This concept of femtosecond pump–probe spectroscopy is the basis for transient electronic absorption spectroscopy (TEAS), a technique used in understanding the photochemistry of UV filters in solution and discussed herein.

#### 1.2.2. Solution-Phase Transient Electronic Absorption Spectroscopy

**Figure 2:**
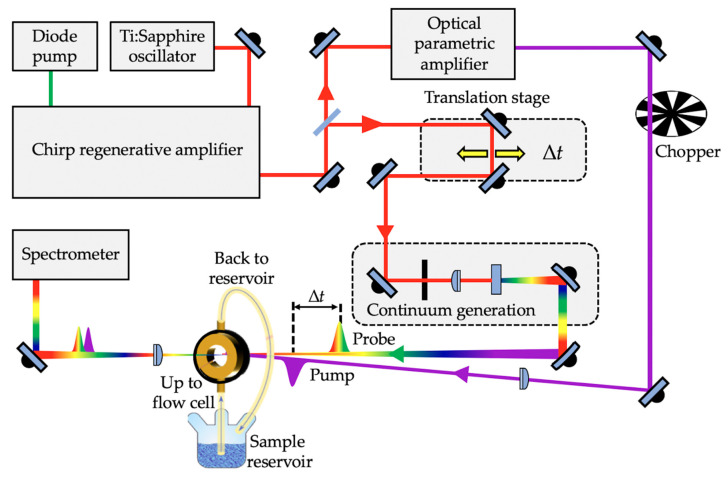
Scheme showing a typical transient electronic absorption spectroscopy (TEAS) experimental setup.

In order to probe the photodynamical processes of a molecule following photoexcitation, time-resolved spectroscopic techniques such as TEAS are often employed [54,91,93,94]; see Figure 2 for a typical TEAS setup. In TEAS, a fraction of the analyte under investigation is photoexcited to a higher lying electronic excited state by an initial femtosecond laser “*pump*” pulse. Thereafter, the photoexcited molecule is monitored with a second laser “*probe*” pulse, typically a white light continuum at a varied time delay (∆*t*) with respect to the pump pulse. The difference between the absorption spectrum of the analyte in the excited state and ground state is measured as changes in the optical density (∆OD), and is calculated as the logarithmic quotient of the transmitted light by the analyte before and after photoexcitation for each probe wavelength (*I*_0_(*λ_probe_*) and *I_t_*(*λ_probe_*, Δ*t*), respectively), expressed mathematically as [93];

(1) $$\Delta \mathrm{OD}\;\left(\lambda_{probe},\;\Delta t\right)=log_{10}\;\left[\frac{I_{0}\left(\lambda_{probe}\right)}{I_{t}\left(\lambda_{probe},\;\Delta t\right)}\right]$$

Ultrafast pulse generation in a TEAS experimental setup is commonly achieved by a Ti:Sapphire oscillator together with regenerative amplification to generate a fundamental 800 nm femtosecond laser pulse with (typically) a pulse energy of a few mJ [83,95]. The laser system output is then split into two beams, allowing for the generation of the pump and probe pulses. The pump pulses are typically generated through optical parametric amplification of the 800 nm beam to achieve tuneable laser pulses across a range of wavelengths (usually the UV-A/UV-B in sunscreen applications). The probe pulses, on the other hand, are generated by focusing the 800 nm beam onto, for example, a calcium fluoride (CaF_2_) window to generate the aforementioned white light continuum [93,96]. The time delay between the pump and probe is typically achieved by delaying the white light through an optical delay line comprising of mirrors and a retroreflector mounted on a precision motorized translation stage. The motorized retroreflector enables the Δ*t* to be varied (through forward and backward travel). The pump and probe pulses are then spatially overlapped and focused into a sample holder containing the analyte under investigation. As shown in Figure 2, a flow cell that allows sample recirculation is often used as the sample holder. This ensures that each TEAS data point is collected from fresh sample, thereby avoiding the photoexcitation of any potential laser-induced photoproducts that might be formed instead of the original form of the molecule under study. Following this, the transmitted probe beam is collimated and sent into a spectrometer. In most cases, to eliminate polarisation effects, the pump pulse polarisation is set at the magic angle of 54.7^o^ with respect to the probe pulse [97]. The spectrum collected at each ∆*t* enables detailed understanding of the dynamical processes of the analyte.

#### 1.2.3. Photophysical and Photochemical Processes

The resultant spectrum from a TEAS experiment is a convolution of the ∆OD signals generated by four main photophysical processes. These photophysical processes can be divided into two categories—those that generate a positive ∆OD and those that generate a negative ∆OD. Detailed explanations of the photophysical processes and how they arise can be found in the review by Berera et al. [93]. However, a brief overview is given here to aid the understanding in Section 2 of this review.

The first photophysical process that generates a negative signal is known as a ground-state bleach (GSB). The pump pulse causes a fraction of molecules to be promoted to their electronic excited state, leading to a depletion in the number of molecules in the electronic ground state. Thus, a greater intensity of probe light reaches the detector for the excited analyte compared to the non-excited analyte. In terms of Equation (1), *I*_0_ < *I_t_* and this generates a negative ∆OD. A GSB has the same spectral signatures as the ground state absorption profile. The second photophysical process that generates a negative signal is stimulated emission. This is the result of a photon from the probe pulse interacting with a molecule in the electronic excited state causing the molecule to return to its electronic ground state. While doing so, an additional photon of the same wavelength is emitted. There is a population of analyte in its electronic excited state and therefore stimulated emission is possible. As above, *I*_0_ < *I_t_* and this generates a negative ∆OD. Stimulated emission occurs around the same spectral region as the fluorescence profile of the molecule and can therefore be easily identified.

The first photophysical process that manifests as a positive signal is an excited-state absorption (ESA). An ESA occurs because of a previously unavailable transition which is now possible from the photoexcited state. Probe wavelengths corresponding to this transition will be absorbed by the excited analyte and not by the non-excited analyte, therefore in terms of Equation (1), *I*_0_ > *I_t_* generating a positive ∆OD. Finally, the molecule’s photochemical relaxation pathway may not result in returning to its original electronic ground state and instead lead to population of a different electronic state (e.g., triplet state) or result in the formation of a photoproduct (e.g., geometric isomer). The resultant triplet state/photoproduct will absorb at different probe wavelengths to the original molecule of interest, and this results in a reduced intensity of light reaching the detector at this particular wavelength for the excited analyte compared to the non-excited analyte. On substitution into Equation (1), a positive ∆OD is produced. Each of these processes contributes towards the overall transient absorption spectra (TAS) that will be presented throughout Section 2 of this review.

Dynamical lifetimes associated with the photophysical and photochemical processes in the TEAS experiment described above can provide valuable information when deducing the photoprotective mechanisms of UV filters. In order to extract these dynamical lifetimes, a global fitting procedure is often employed. Detailed explanations of this and other fitting methods have been reported in previous literatures [98,99,100]. Furthermore, a comprehensive review on the fitting procedure for time-resolved spectroscopy experiments has been recently reported [101].

## 2. Case Studies

### 2.1. Plant-Inspired Ultraviolet Filters

As detailed in Section 1.1.2, the drawbacks surrounding the organic UV filters currently used in sunscreen formulations are motivating the research for nature-inspired photoprotection that is both human- and eco-friendly [102,103]. Nature-inspired UV filters from plants previously studied for their photoprotection mechanism will be reviewed in this section. In this review, we classify the reported studies of plant-derived UV filters into three groups: SM and SA (Section 2.1.1), sinapate ester derivatives (Section 2.1.2), and a symmetrically functionalized sinapate ester (Section 2.1.3). The associated findings with these groups of plant-derived UV filters are discussed in detail below.

#### 2.1.1. Sinapoyl Malate and Sinapic Acid

SM, an ester derivative of SA, shown in Figure 1a, has been previously identified as the UV-absorbing molecule preventing the adverse effects of overexposure to UV-B radiation in *Arabidopsis* plants [12,52,104]. Dean et al. [53] were the first to report laser spectroscopy work on plant UV filters, and although their work was carried out using vibrationally resolved UV spectroscopy, it has since served as the motivation to many of the reported time-resolved laser spectroscopy studies of plant UV filters.

**Figure 3:**
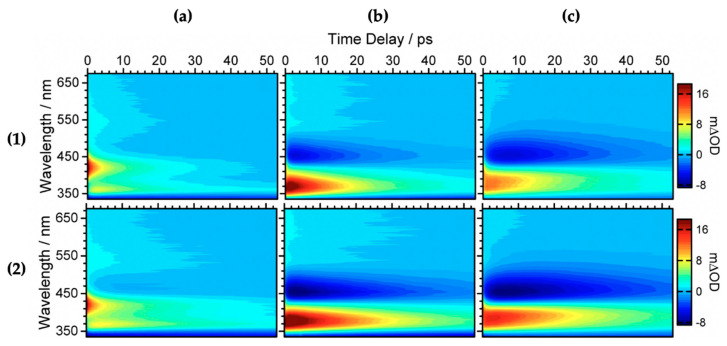
Transient absorption spectra (TAS) displayed as false colour maps of (1) SA and (2) SM in (**a**) dioxane, (**b**) acetonitrile and (**c**) methanol, respectively. Reproduced and adapted with permission from [104], licensed under CC-BY. © 2015 American Chemical Society.

Baker et al. [104] investigated the relaxation mechanism of SM and SA using time-resolved TEAS in a variety of solvents (dioxane, acetonitrile and methanol) to explore their photochemistry in bulk solution and also to study the effects of solvent polarity on the excited-state dynamics. Baker et al.’s experimental TAS revealed that solvent-dependent TAS with similar excited-state features were observed for both SM and SA (presented in Figure 3) following photoexcitation at their respective absorption maxima (λ_max_) in dioxane, acetonitrile and methanol [104]. For SM and SA in dioxane, the TAS reported by Baker et al. [104] are dominated by three features: firstly, an initial intense absorption centred at ~420 nm assigned to ESA which decays away to baseline by ~50 ps; a second ESA covering the spectral region of ~420–650 nm which decays to baseline by ~20 ps; and, finally, a negative signal below ~350 nm which was assigned to GSB. The GSB does not fully recover at the maximum available pump probe time delay of 2 ns. In the same way, the TAS of SM and SA in acetonitrile and methanol are also dominated by the three features observed in the TAS of SM and SA in dioxane, but with the ESA blue shifted, centred at ~370 nm instead of the ~420 nm seen in dioxane. In addition, a strong negative feature centred around ~460 nm was assigned to stimulated emission.

Furthermore, the intense positive signal centred at ~370 nm observed in both the continuous-wave UV irradiation difference spectrum (∆UV/visible spectrum) and the TAS at ∆*t* = 2 ns in all solvent environments is attributed to a long-lived photoproduct. The authors assigned this feature to (in part) the *cis* isomer formed following UV absorption in both SM and SA [104]. However, the spectrum is not reproduced here.

The authors employed a global fitting procedure to obtain quantitative insight into the dynamical processes of the reported TAS. Detailed information on this fitting procedure has been reported in separate publications [98,105]. It was found that SM and SA could be accurately fitted with three time constants (*τ_n_*) in all the solvents. These are shown in Table 1.

**Figure 4:**
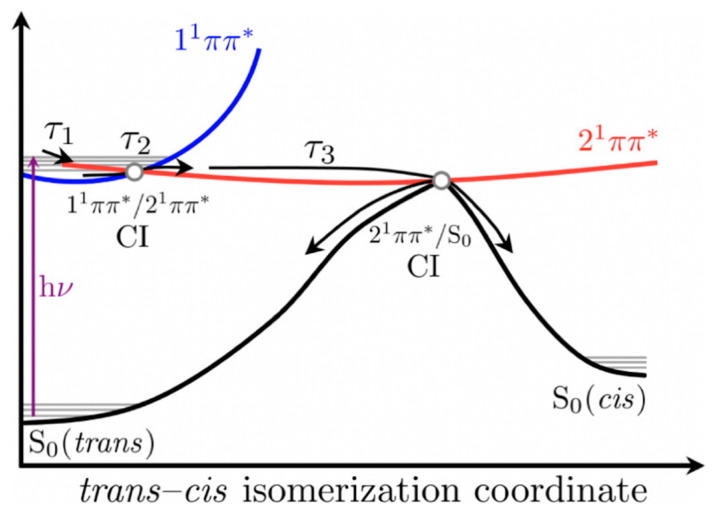
Relaxation scheme of SM and SA proposed by Baker et al. [104] Reproduced and adapted with permission from [104], licensed under CC-BY. © 2015 American Chemical Society.

The authors proposed that following photoexcitation to the first singlet excited state (1^1^ππ*), SM and SA undergo multiple processes that are convoluted together and defined by *τ*_1_ and *τ*_2_ presented in Table 1, thereby making the distinct assignment of any one process with these lifetimes difficult. Nonetheless, Baker et al. [104] proposed that *τ*_1_ defines the evolution out of the Franck–Condon window and *τ*_2_ was suggested to have resulted from the solvent rearrangement and internal conversion via a 1^1^ππ*/2^1^ππ* conical intersection (CI). Finally, *τ*_3_ was assigned to isomerisation along the 2^1^ππ* state to generate either the *cis* or *trans* isomers in their respective S_0_ through a 2^1^ππ*/S_0_ CI. A summary of the relaxation dynamics is described in the scheme shown in Figure 4 [104]. An alternative relaxation mechanism was proposed without recourse to the 2^1^ππ* intermediary state. In this case, the authors suggest that *τ*_1_ and *τ*_2_ correspond to the excited-state population relaxing along the S_1_ potential energy surface (PES) before relaxing back to S_0_ via 1^1^ππ*/S_0_ CI on the *τ*_3_ timescale.

To summarize, the results of the study by Baker et al. [104] showed a favourable, ultrafast relaxation mechanism for SM and SA following excitation. This highlights the ability of these systems to efficiently dissipate the absorbed UV energy and bypass potentially deleterious processes (triplet state, radicals, etc.), which is a positive attribute for a UV filter in sunscreen formulation.

**Figure 5:**
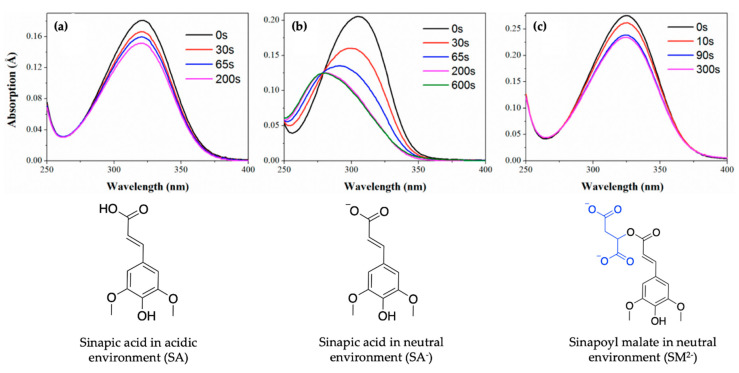
UV/visible spectra showing the photoisomerisation of (**a**) SA, (**b**) SA^−^ and (**c**) SM^2−^, with the group added to SA shown in blue, at varying duration of pulsed irradiation. Corresponding structures are shown under UV/visible spectra. Reproduced and adapted with permission from [106]. © 2017 American Chemical Society.

Baker et al.’s work suggests that SM and SA undergo similar photodynamics following photoexcitation [104]. However, no dynamical behaviour suggests the choice of SM (e.g., in thale cress) as the plant UV filter over SA. In an attempt to justify nature’s choice of SM and not SA as the UV filter in plants, Luo et al. [106] studied the ionic forms of SM and SA (SM^2−^ and SA^−^), as they occur under physiological pH conditions in plant leaves, using a combination of TEAS and time-dependent density functional theory (TD-DFT). The results of their study revealed that SM^2−^, SA^−^ and SA undergo a non-radiative decay via a barrierless *trans–cis* photoisomerisation relaxation pathway leading to the formation of *cis* photoproducts. This compares well with the proposed relaxation mechanism for SM and SA by Baker et al. [104] even when pH is taken into account. Nevertheless, Luo et al. [106] reported that following continuous irradiation (i.e., static irradiation of the sample with continuous-wave beam), the photoisomerisation of the *trans*-SM^2−^ isomer (and *trans*-SA) yields the *cis* isomer with absorption spectrum characteristics similar in intensities and shape to the *trans* isomer spectrum, as shown in Figure 5a,c. As evident in Figure 5b, the photoisomerisation of *trans*-SA^-^ results in a *cis* absorption spectrum that is significantly blue shifted and decreased in intensity following irradiation, suggesting a change in spectral characteristics. Luo et al. [106] concluded that the similarity between the spectral properties of *trans*- and *cis*-SM^2−^ following photoisomerisation suggests SM^2-^ as a better UV filter and could justify nature’s selection of SM rather than SA as plants UV filter [106].

A further study by Horbury et al. [107] has investigated SM more closely to explore the influence of solvent viscosity on the proposed photodynamics [107]. A comprehensive review of this study has been reported in a previous publication [108]. Briefly, following TEAS measurements, the study revealed that the time constant assigned to the relaxation of the excited-state population along the *trans–cis* photoisomerisation coordinate (*viz. τ*_2_ in SM and SA, supra) increases considerably with increasing viscosity, from 47 ps in ethanol to 560 ps in glycerol. The authors concluded that photoisomerisation of SM is likely facilitated by the out-of-plane rotation about the C=C bond (i.e., a large-amplitude vibrational motion) and consequently is the reason for the large solvent viscosity effect on its photodynamics [107].

The aforementioned studies not only provide insight into understanding the photodynamics of SM and SA, but they also provide information about the solvent environments (pH and viscosity) and the justification of plant selection of SM over SA for photoprotection. Furthermore, the ultrafast excited-state lifetime and the photoisomerisation relaxation mechanism suggest that an appropriate chemical modification of SM and SA could make a good UV filter for use in sunscreen formulations provided it has no adverse effects on the skin and ecosystem.

#### 2.1.2. Sinapate Ester Derivatives

Building on the reported photodynamics for SM and SA (Section 2.1.1), various sinapate ester derivatives that bridge the chemical complexity between these two have been studied in an attempt to design efficient plant-based UV filters for sunscreen formulation. TEAS and TD-DFT studies have been reported for methyl sinapate (MS), isopropyl sinapate (IS), sinapoyl methyl lactate (SML) and sinapoyl dimethyl malate (SDM) in various solvents and poly(vinyl alcohol) hydrogel film [104,109,110]. The results of these studies have been reviewed in more detail in previous publications [54,55]. Briefly, the results of the study carried out on MS, IS, SML and SDM in buffer solution (0.1 M NaH_2_PO_4_, 0.1 M Na_2_HPO_4_, pH = 6.8) by Liu et al. [109] revealed that these sinapate esters undergo relaxation dynamics along the *trans–cis* isomerisation coordinate, as seen in SM and SA [104,106]. Additionally, the extracted time constant from the fit of the data had non-linear dependence on the size of the sinapate ester (in size order MS < IS < SML < SDM). Liu et al. [109] then took an additional step towards mimicking the effect of applying a sunscreen to the surface of a skin by including the sinapate esters within a poly(vinyl alcohol) (PVA) hydrogel film. The authors justified the inclusion of PVA as a skin model due to its complex hydrogen bonding network [109]. The authors observed a 25-fold increase in the time taken for isomerisation of the sinapate esters to occur in PVA following photoexcitation compare to the values observed in buffer solution. The increased time constant for isomerisation in PVA is evidence of the restriction of motion in the film environment. This work presents the need to study plant-based UV filters in a close-to-real environment.

**Figure 6:**
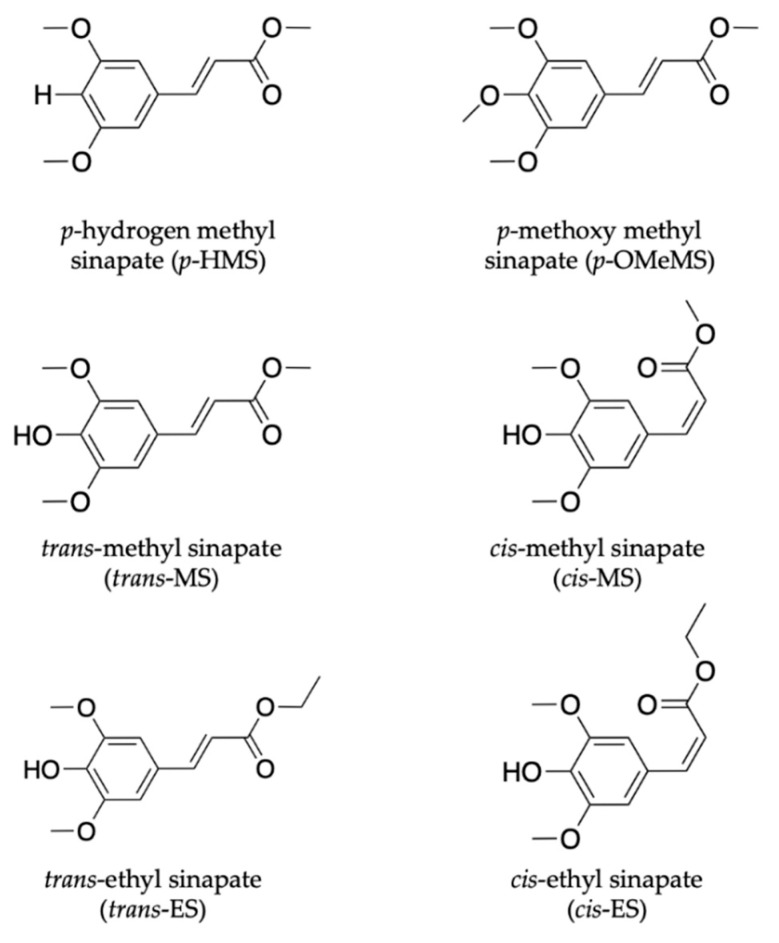
Chemical structures of sinapate ester derivatives including *p*-hydrogen methyl sinapate (*p*-HMS), *p*-methoxy methyl sinapate (*p*-OMEMS), *trans*-methyl sinapate (*trans*-MS) and *cis*-methyl sinapate (*cis*-MS) studied by Zhao et al. [111,113]. Also shown are *trans*-ethyl sinapate (*trans*-ES) and *cis*-ethyl sinapate (*cis*-ES) studied by Horbury et al. [114].

In the work by Zhao et al. [111], the effect of *para* substituents on the excited-state photodynamics of MS (the *trans*- form) including *para* methoxy methyl sinapate (*p*-OMeMS) and *para* hydrogen methyl sinapate (*p*-HMS) shown in Figure 6 were investigated. Briefly, the findings revealed that *p*-OMeMS and MS undergo similar dynamics along the *trans–cis* photoisomerisation coordinate, as seen in previous studies [104,109,110]. *p*-HMS, on the other hand, undergoes different dynamics with an internal conversion from the initial optically bright excited state (denoted V(ππ*)) to a relative dark state (denoted V’(ππ*)), which results into branching of the excited-state relaxation. While the V(ππ*) still relaxes non-radiatively, as seen in MS and *p*-OMeMS, the V’(ππ*) decays to the ground state via fluorescence (i.e., emission of a photon) in 5 ns [111]. The radiative decay reported for the relaxation mechanism of *p*-HMS in the excited state is undesirable of a UV filter; it could result in the formation of harmful photoproducts (including radicals), which implies that it is not a good candidate for inclusion in a sunscreen [54]. On the other hand, the favourable energy dissipation mechanism of *p*-OMeMS and MS suggests the contrary for these two systems. However, the potential genotoxicity concerns surrounding the *cis* isomer (photoproduct), as reported for related cinnamates [112], need to be further explored. This study distinguishes the difference between the dynamics mediated via the V(ππ*) and V’(ππ*) states and revealed the importance of substituents in the molecular design of plant-based UV filters.

**Figure 7:**
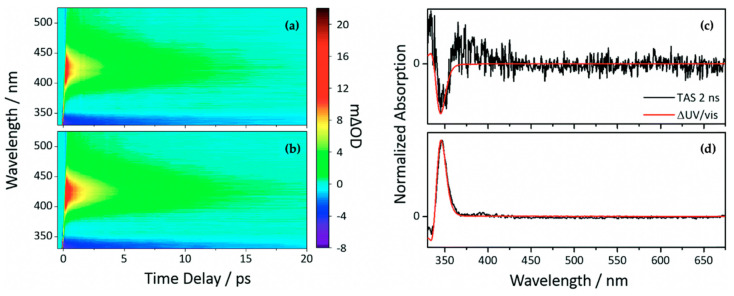
TAS displayed as false colour maps of (**a**) *cis*-ES and (**b**) *trans*-ES in cyclohexane photoexcited at 319 nm. Plot to show the TAS taken at ∆*t* = 2 ns of (**c**) *cis*-ES and (**d**) *trans*-ES overlaid with ∆UV/visible spectra. Reproduced and adapted with permission from [114], licensed under CC BY 3.0. Published by the Royal Society of Chemistry.

Another class of sinapate ester that has been recently studied to elucidate its photodynamics and photoprotection mechanisms is ethyl sinapate (ES). Horbury et al. [114] investigated the photochemistry of the *cis* and *trans* isomers of ES, shown in Figure 6, in cyclohexane using TEAS in an attempt to understand the isomer-specific photoprotection of plant UV filters. The TAS of both isomers shown in Figure 7a,b are dominated by two features, an ESA centred at ~425 nm and a GSB centred at ~335 nm. From their data, Horbury et al. [114] establish that at pump–probe delay times (∆*t*) > 20 ps, the ESA in the TAS has decayed to zero ∆OD (where ∆OD denotes the change in optical density). However, at longer ∆*t*, the TAS of both isomers revealed a pair of spectral features in the probe region of 330–350 nm assigned to GSB and photoproduct absorption. The authors determined from the TAS at ∆*t* = 2 ns, shown in Figure 7c,d, that this pair of features differ between the two isomers. In the *trans* isomer, the GSB is centred at ~335 nm, while the photoproduct absorption is centred at ~345 nm. For the *cis* isomer, the reverse was observed, with the GSB at ~345 nm and photoproduct absorption at ~335 nm. Horbury et al. [114] identified the species responsible for the photoproducts by comparing the TAS at ∆*t* = 2 ns to the steady-state difference spectra (∆UV/visible spectra), an approach that has been previously used by Baker et al. [104] in their study of MS. The similarities between the TAS and ∆UV/visible spectrum (see Figure 7c,d) for photoexcited *trans*-ES led the authors to propose that the *cis* isomer is being formed, while the TAS and ∆UV/visible spectrum for initially photoexcited *cis*-ES revealed that the *trans* isomer is formed.

Following qualitative analysis of the data, the quantitative insight into the dynamical process of the TAS was obtained by sequential ($A\overset{\tau_{1}}{\to }B\overset{\tau_{2}}{\to }C\overset{\tau_{3}}{\to }D\ldots$) global fitting following the procedure previously reported [54,98,99,105]. It was reported that the excited-state dynamics of both isomers could be fitted with three time constants shown in Table 2. The extracted time constants *τ*_ivr_ and *τ*_iso_ for *trans*-ES were assigned to intramolecular vibrational redistribution (IVR) and photoisomerisation along the *trans* to *cis* isomerisation coordinate, respectively. The authors reported that following this (isomerisation) coordinate, the formation of *cis*-ES as a photoproduct and the initially photoexcited *trans*-ES were observed, both in their electronic ground state, with the lifetime *τ*_pp_. Their evidence for these assignments was based on the dynamics of the *trans* isomers of other sinapates, previously discussed in the literature [104,110]. Seeing the similarities between the TAS and extracted time constants between *cis*-ES and *trans*-ES, the authors proposed that *cis*-ES undergoes the same photodynamics as *trans*-ES, with the photoisomerisation being *cis* to *trans* instead and *τ*_pp_ denoting the lifetime of *trans*-ES as the photoproduct and initially photoexcited *cis*-ES in their electronic ground state. However, from their results, the authors observed that photoisomerisation of *trans*-ES occurs 10% faster compared to *cis*-ES, and there is more recovery of the *cis*-ES GSB than the GSB of *trans*-ES, thereby suggesting that the isomerisation is biased towards the *cis*-isomer. This claim is further supported from the extracted ratio of *cis*- to *trans*-ES following steady-state irradiation, which showed a 70:30 ratio of *cis* to *trans* isomer irrespective of the starting isomer. The authors concluded that the resultant ratio is kinetically rather than thermodynamically driven.

Overall, it was determined that the photochemistry of the *cis* isomer is very likely comparable to the photochemistry of the naturally synthesized *trans* isomer, in terms of dissipating the dangerous excess energy imparted due to potentially phototoxic UV absorption. Further, the efficient ultrafast energy dissipation mechanism along the *trans–cis* isomerisation coordinate resulting in the repopulation of the ground state implies that there is less chance for toxic relaxation pathways (triplet state, radicals, etc.). This has important implications in sunscreen application.

**Figure 8:**
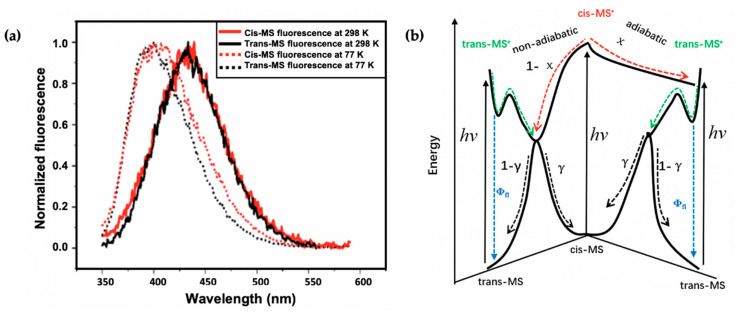
(**a**) Steady-state emission spectra of *trans*-MS (black solid line) and *cis*-MS (red solid line) at room temperature. The corresponding emission spectra at 77 K are shown as dotted lines. (**b**) Schematic of the proposed relaxation scheme of MS by Zhao et al. [113]. Reproduced and adapted with permission from [113]. © 2019 American Chemical Society.

Recently, Zhao et al. [113] took the study of Horbury et al. [114] a step further by combining fluorescence spectroscopy, TEAS and quantum chemical calculations to determine the photoprotection mechanisms of the *trans* and *cis* isomer of MS (see Figure 6). The observed shapes and peaks of the fluorescence emission of *trans*-MS and *cis*-MS are similar at room temperature (see Figure 8a). However, the fluorescence quantum yield reported for *trans*-MS is about 2.5 times larger than that of *cis*-MS. The authors concluded that the smaller quantum yield of *cis*-MS resulted from the higher rate of non-radiative decay compared to *trans*-MS [113]. At 77 K, the emission spectra of the two isomers differ compared to those observed at room temperature (see Figure 8a). Zhao et al. [113] concluded that the temperature-dependent emission behaviour of the two isomers is an indication that the fluorescence emission of *cis*-MS may result from excited *trans*-MS*, which implies that adiabatic *cis*-MS* → *trans*-MS* isomerisation has taken place [113]. Furthermore, a similar isomerisation barrier energy was estimated for both *trans*-MS and *cis*-MS (i.e., 2.7 and 2.9 kcal/mol, respectively) on the PES from the temperature-dependent fluorescence quantum yield [113]. Combining the isomerisation barrier and temperature-dependent emission results, the authors concluded that the isomerisation barrier of *cis*-MS stems from the *trans*-MS in an excited state.

In addition to the previously reported relaxation mechanism in MS [104,109,110,111], the findings revealed that for the thermally stable *trans*-MS, the S_1_/S_0_ CI is reached on the excited PES after a barrier is overcome along the allylic C=C double bond. This then facilitates the non-adiabatic relaxation to the ground state, generating either the original *trans*-MS or a *cis*-MS photoproduct. Contrary to *trans*-MS, the relaxation pathway of the *cis*-MS includes decay of the excited *cis*-MS on a sub-picosecond timescale, followed by the decay of the *trans*-MS formed adiabatically from the excited *cis*-MS [113]. Consequently, the authors conclude that the isomerisation barrier of the *cis*-MS* stems from the isomerisation barrier for the allylic C=C double bond twisting of trans-MS*. Combining these results, Zhao et al. [113] claim that the relaxation mechanism of *cis*-MS includes an adiabatic relaxation pathway competing with a non-adiabatic relaxation pathway. A summary of the relaxation dynamics is shown in Figure 8b. This work revealed the difference between the photodynamics of *cis*- and *trans*-MS. 

#### 2.1.3. Symmetrically Functionalized Sinapate Ester

**Figure 9:**
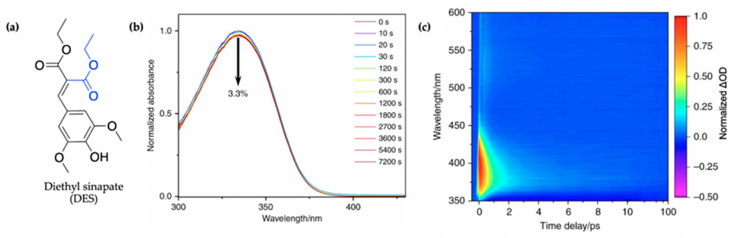
(**a**) Structure of diethyl sinapate (DES) with the group added to the monoester precursor of ES shown in blue. (**b**) Long-term photostability of DES. UV/visible spectra of DES in C12-C15 alkyl benzoate, at varying durations of irradiation at 335 nm and replicating solar intensity. (**c**) Normalized TAS displayed as a false colour map of DES in C12-15 alkyl benzoate (AB) deposited on a synthetic skin mimic (termed DES VC/AB), photoexcited at 335 nm. The timescale is plotted linearly from −0.5 to 10 ps, then as a log scale from 10 to 100 ps. Reproduced and adapted with permission from [115], licensed under CC BY 4.0. Published by Springer Nature.

To circumvent the concerns surrounding the potential genotoxicity of the *cis* isomer formed in sinapate esters following UV absorption, as seen in related cinnamates [112], Horbury et al. [115] studied a symmetrically functionalized sinapate ester. This was achieved by adding identical ester moieties across the allylic double bond in previously studied ES [114], resulting in diethyl sinapate (DES) with identical *trans* and *cis* isomers shown in Figure 9a. Using TEAS, the photodynamics of this novel sinapate ester was studied in an industrial standard emollient (C12-15 alkyl benzoate, moisturizer commonly used in commercially available sunscreens) and on a synthetic skin mimic, VITRO-CORNEUM^©^ (VC) providing a closer-to-real-life application environment for sunscreens. In addition to the TEAS measurements, steady-state UV/visible, endocrine disruption, and antioxidant properties of DES are reported in their study. The results of this study are discussed herein.

In their steady-state UV/visible spectra shown in Figure 9b, Horbury et al. [115] observed a red-shifted spectrum compared to the monoester (ES), the cause of which was assigned to the additional π-system conjugation in DES. Furthermore, the authors observed improved photostability, with a 3.3% reduction in absorption for DES following 2 h of irradiation (see Figure 9b), compared to 16% previously reported for ES after 45 min of irradiation [114]. The cause of this improved photostability was attributed to needless photoequilibrium between the *trans* and *cis* isomers of DES following photoexcitation [115]. 

The TAS of DES in alkyl benzoate after being applied to the surface of VC (termed DES VC/AB) shown as a false colour map by the authors is presented in Figure 9c. For comparison, additional TAS of DES were also collected in C12-15 alkyl benzoate only (termed DES/AB), ethanol and cyclohexane to provide a range of solvent environments. However, these are not reproduced here. Similar to what has been seen in ES [114], the TAS of DES VC/AB consists of a single ESA which quickly decays within a time delay (∆*t*) of <100 fs as the excited-state population evolves out of the Franck–Condon region. In ES, this feature was assigned to geometry relaxation, the authors suggest that DES undergoes a similar mechanism. Following the geometry relaxation, the three notable spectral features that ensues in the TAS reported by Horbury et al. [115] are a GSB at ~350 nm, a strong ESA at ~380 nm and a second weaker ESA at ~540 nm, all of which are in accordance with what has been observed in the literature for ES [114]. At ∆*t* = 4 ps, the ESA observed in the TAS at a probe wavelength of ~540 nm has decayed to zero ∆OD while a remnant of the GSB and ESA at ~380 nm remains. In previous studies, these have been attributed to a phenoxy radical or isomer formation. However, the authors ruled out isomer and radical formation due to the identical *cis* and *trans* isomer and decay of the feature over time.

Again, quantitative insight into the TAS features and dynamics was attained using global fitting. This paper reports the kinetics in terms of rate constants compared to its inverse of time constants used to describe the kinetics for other systems reviewed herein. It was found that all datasets, DES VC/AB, DES/AB, and DES in ethanol, could be fitted with five kinetic rate constants (*k*_1_–*k*_5_) except for DES in cyclohexane which returned three rate constants. These rate constants are shown in Table 3. The authors assigned the dynamics of *k*_1_-*k*_3_ based on previously reported literature for ES [114] and other sinapate esters [104,114]. The rate constant *k*_1_ represents geometry relaxation out of the Franck–Condon region, *k*_2_ defines evolution along the excited state including vibrational cooling and solvent rearrangement and *k*_3_ describes the rate constant for photoisomerisation and repopulation of the electronic ground state. However, the authors highlighted that since DES is symmetrical, it is impossible to determine whether the photoisomerisation occurs completely or is an aborted photoisomerisation. Additional *k*_4_ observed for DES was assigned to a ^1^nπ* state which has been identified to play a role in cinnamate photodynamics. Finally, *k*_5_ defines a long-lived GSB resulting from either a molecular photoproduct or a trapped excited-state population [115].

Even though the authors observed similar photochemistry for DES in all the solvents, the differences associated with the rate constants were highlighted, with a focus on comparison between DES VC/AB and DES/AB. This was done to see whether the more realistic “skin environment” had any effect on the dynamics of DES. Briefly, the authors observed an almost three-fold increase in *k*_4_ for DES/AB compared to DES VC/AB. This difference was attributed to a potential greater barrier experienced by population trapped in the excited state towards ground-state recovery in DES VC/AB. 

Additional endocrine disruption measurements of DES revealed no adverse effect to alpha estrogen receptor (ERα) or the xenobiotic receptor (PXR). This is promising, since most commercially available organic UV filters have been reported to act as endocrine disruptors and causing severe health effects [116]. Similarly, the antioxidant potential measurements revealed that DES can act as antioxidant, lending additional benefits to the inclusion in sunscreen formulation. 

To summarize, Horbury et al. [115] have built on previous works conducted on sinapate esters in an attempt to circumvent concerns around genotoxicity of potential photoproducts resulting from *trans–cis* photoisomerisation. The red-shifted UV-A absorption of DES is promising due to the sparsity of FDA/EU-approved UV-A filters. Although consistent photodynamics were observed between DES in an emollient used in commercial sunscreen formulation, and when deposited on VC, the mild dependence of DES photodynamics on its environment highlights the need to study potential UV filters in a closer-to-real-life setting.

This work showed that sinapate esters with symmetric molecular functionality could be promising towards achieving efficient UV filters in the UV-A region. However, further investigation is needed in order to identify beneficial modifications that may facilitate ultrafast dynamics without leaving long-lived photoproducts on skin. Importantly, the non-radiative, ultrafast energy dissipation mechanism observed and the extended photostability, following two hours of steady-state irradiation, present opportunities for the potential inclusion of DES in future sunscreen formulation.

In summary, the works reported to date on the studies of plant-inspired UV filters (sinapate esters) revealed that this class of molecules undergoes an efficient, non-radiative energy dissipation pathway following photoexcitation. That being said, a long-lived, and potentially toxic, photoproduct identified as the isomer of the initial form of the ester is associated with sinapate esters; appropriate modification of the chemical structure such as symmetric functionalisation, as shown in the works of Horbury et al. [115], could circumvent this photoproduct formation. This provides important opportunities for the potential application of sinapate esters in sunscreens. However, further complementary works on the biological safety, including studies to assess endocrine disruption and photocontact dermatitis, would provide additional insight into their potential use in commercial sunscreen formulations.

### 2.2. Microbial-Inspired Ultraviolet Filters

As previously highlighted in Section 1.1.5, MAAs are either composed of a cyclohexenone or cyclohexenimine core; see Figure 1b. This section of the review will evaluate the computational and experimental work conducted to date on each core component. For the purpose of the remaining sections of this review, we will revert to referring to molecules with a cyclohexenone core as being mycosporines or mycosporine motifs and molecules with a cyclohexenimine core as mycosporine-like amino acids (MAAs) or MAA motifs.

#### 2.2.1. Mycosporines

**Figure 10:**
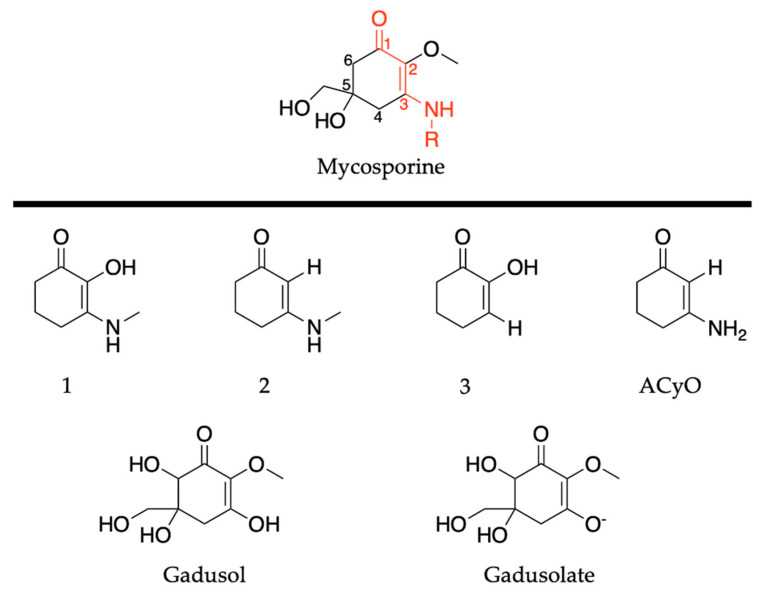
Structures of mycosporine motifs studied by Losantos et al. [89] and Woolley et al. [85], and gadusol/gadusolate studied by Losantos et al. [87].

Mycosporines are composed of a cyclohexenone core, with a carbonyl at carbon one, a methoxy group attached to carbon two, and hydroxy and hydroxymethyl substituents at carbon five; see Figure 10. Carbon three is substituted with an amino group (represented as R in Figure 10), and this is what causes the variability among mycosporines [60]. We begin the mycosporine discussion by reviewing work conducted by Losantos et al. [89] and Woolley et al. [85] on basic cyclohexenone structures, herein referred to as mycosporine motifs. Further details of these authors’ works can be found in the reviews by Holt and Stavros [55] and Woolley and Stavros [83]. As a result, only a brief review will be given here. Losantos et al. [89] studied molecules 1–3, shown in Figure 10, employing a high level of theory known as complete active space perturbation to second order (CASPT2) [117] with a complete active space self-consistent field reference wavefunction (CASSCF) [118], herein referred to as CASPT2//CASSCF methodology [119,120]. Further details on the methodology, such as the active space and basis set used, can be found in the original research [89], as is the case with all the theoretical work reviewed in Section 2. Woolley et al. [85] studied a modification of molecule 2, 3-aminocyclohex-2-ene-1-one (termed ACyO, the methyl group on the nitrogen attached to carbon three in molecule 2 is replaced with a hydrogen), experimentally using TEAS along with complementary computational studies; see Figure 10. All four mycosporine motifs are composed of the same cyclohexenone core structure, with varying substituents on carbons two and three.

**Figure 11:**
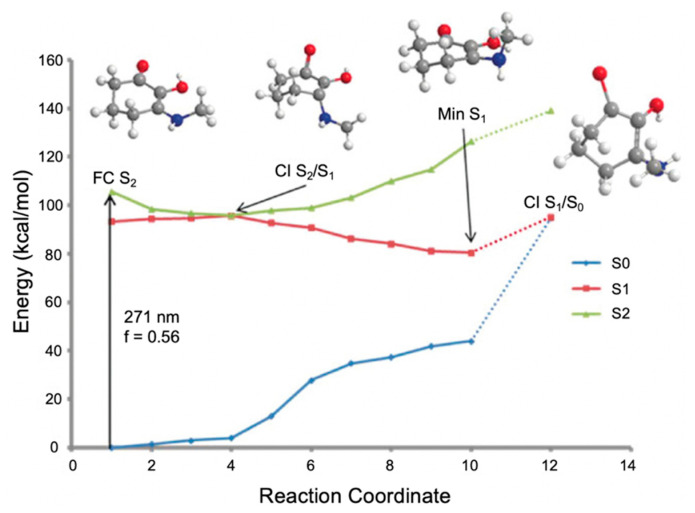
Computed minimum energy path (MEP) of molecule 1 by Losantos et al. [89] where f is the oscillator strength. Reproduced and adapted with permission from [89]. © 2017 Wiley-VCH Verlag GmbH & Co. KGaA, Weinheim.

In the work by Losantos et al. [89], CASPT2//CASSCF methodology [119,120] was used on molecules 1–3 to compute the absorption spectrum, the critical points along the PES and the minimum energy paths (MEPs) connecting them [89]. Similar critical points along all three molecules’ PES were determined. As a result, only molecule 1 was explored in more detail by the authors to elucidate the photoprotection mechanism; Figure 11 presents the MEP for molecule 1 [89]. In brief, photoexcitation leads to populating the S_2_ excited state via a ^1^ππ* transition. An out-of-plane geometry distortion of the substituents at carbons one, two and three results in access to the S_2_/S_1_ CI; see Figure 10 for atom numbers. Relaxation along the S_1_ PES finds a minimum on the S_1_ coordinate with planar geometry. A higher energy S_1_/S_0_ CI was located with the same out-of-plane geometry distortion as was found at the S_2_/S_1_ CI. Losantos et al. [89] concluded that after photoexcitation, molecule 1 would become trapped in the S_1_ minimum and suggested that electronic ground-state recovery would occur via radiative decay. Such a decay mechanism is undesirable of a UV filter, because the emitted photon could be damaging to skin cells [54]. As such, its inclusion in a sunscreen formulation is not ideal. Consequently, Losantos et al. [89] did not investigate these computationally studied mycosporine motifs further through experimental analysis.

TEAS experiments on ACyO were carried out by Woolley et al. [85] in order to understand whether the predicted photoprotective mechanism by Losantos et al. [89] could be observed experimentally. Acetonitrile and methanol were used as solvents to unravel solvent effects on the observed photodynamics. Their findings were that in both solvents, a persistent ESA was present at extended time delays, Δ*t* > 2.5 ns, and this was attributed to the trapped population of ACyO in the minimum of the S_1_ PES [85]. As the TAS have been reviewed previously [55,83], no further details are given here. In addition to the TEAS experiments, Woolley et al. [85] also conducted complementary CASSCF calculations on ACyO to calculate critical points along the S_1_ PES. Their findings were that the slight modification between molecule 2 and ACyO has little effect on the electronic structure. A minimum on the S_1_ PES was calculated with an energy barrier of 0.04 eV for the computed S_1_/S_0_ CI [85].

At this point, there is a strong correlation between the findings of Woolley et al. [85] and Losantos et al. [89] These simple mycosporine motifs seem to display undesirable properties of a UV filter, as efficient repopulation of the electronic ground state on ultrafast timescales is not observed. The long-lived excited state on the S_1_ increases the probability of competing reactive pathways that could result in photoproduct formation. Possible side reactions in commercial sunscreens can then lead to undesirable photoallergic contact dermatitis, a problem cited before with some current UV filters [43,44,45,121,122]. Furthermore, it was predicted by Losantos et al. [89] that molecules 1–3 would decay radiatively to repopulate the electronic ground state. Interestingly, however, Woolley et al. [85] did not observe any fluorescence from ACyO in either methanol or acetonitrile. This was attributed to the poor Franck–Condon overlap between the S_1_ minimum and the electronic ground state in ACyO; relaxation of ACyO to its electronic ground state must therefore occur non-radiatively or go on to form a photoproduct beyond 2.5 ns.

Although the presented work to date has implied that mycosporine motifs do not display the ideal properties of a UV filter, work on natural mycosporines and related molecules, such as gadusol shown in Figure 10, suggest otherwise [81,82,87,123]. The studies on gadusol have been reviewed in a previous publication [62], so only a brief overview is given here. The 4-deoxygadusol (gadusol with a H at carbon 6; see Figure 10) is often the precursor molecule in the biosynthesis of mycosporines [58]. At biological pH, gadusol is present as its conjugate anion gadusolate; see Figure 10. The absorption maxima of the two species is red shifted ~30 nm for gadusolate and corresponds to a ^1^ππ* transition [82,87,124]. Arbeloa et al. [82] conducted several experimental studies on gadusol and gadusolate to determine the photostability and deactivation pathways of these species. The photodecomposition quantum yields were determined as 3.6 × 10^−2^ and 1.4 × 10^−4^ for gadusol and gadusolate, respectively, in aqueous solutions. The authors conclude that although both species demonstrate good photostability, it is apparent that gadusolate dissipates its absorbed energy quicker than gadusol, reducing the probability of competing reactive relaxation pathways. Additionally, no fluorescence was observed for either gadusol or gadusolate, providing evidence for non-radiative decay upon photoexcitation in both species [82].

To evaluate the decay mechanism for gadusolate, Arbeloa et al. [82] performed photoacoustic calorimetry, which is a technique that can provide a quantitative measurement for the non-radiative dissipation of a solute’s energy after photoexcitation [125,126,127]. The experimental setup and detailed explanations on photoacoustic calorimetry can be found in the following reviews: Braslavsky and Heibel [125], Arnaut et al. [126] and, Gensch and Viappiani [127]. For a base understanding, it is important to highlight that in a photoacoustic calorimetry experiment, a calorimetric standard is used that does not undergo any radiative decay pathways and absorbs at a similar wavelength to the solute [128]. In the case of gadusolate, the ratio of photoacoustic signal amplitude between gadusolate and the calorimetric standard was equivalent within the experimental error, indicating that gadusolate dissipates its absorbed energy as heat within the first tens of nanoseconds [82]. The above findings indicate that repopulation of the ground state via non-radiative decay mechanisms occurs for both gadusol and gadusolate, which in turn provides some evidence for nature’s choice to use mycosporines as effective UV filters.

**Figure 12:**
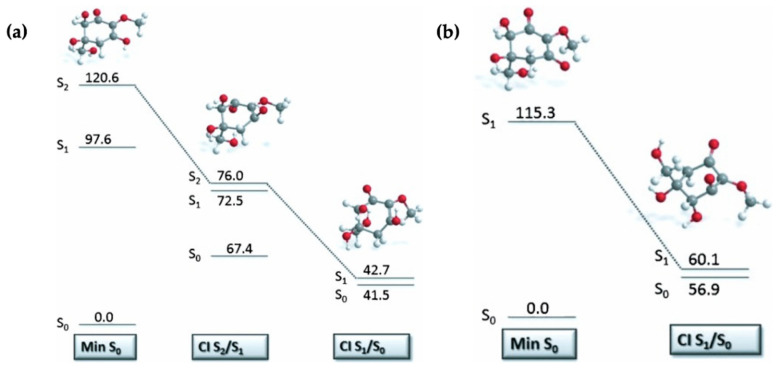
Critical points along the MEP for (**a**) gadusol and (**b**) gadusolate computed by Losantos et al. [87] where the energies are reported in kcal mol^−1^ relative to the electronic ground-state minimum. Reproduced and adapted with permission from [87], licensed under CC BY-NC-ND 4.0. © 2015 The Authors. Published by Wiley-VCH Verlag GmbH & Co. KGaA.

Losantos et al. [87] evaluated the MEPs of gadusol and gadusolate using CASPT2//CASSCF methodology [119,120] in a similar way to the mycosporine motifs [89] discussed above. The critical points along each MEP are displayed in Figure 12 to illustrate the proposed mechanisms for each species by Losantos et al. [87] Upon photoexcitation, it was found that gadusol is promoted to the optically bright S_2_ excited state, via a ^1^ππ* transition. At the S_2_/S_1_ CI, an out-of-plane movement of the oxygen atoms of the C=O and OH moieties within the chromophore is present along with elongation of the C=C and C=O bonds. Additionally, compared to the S_0_ minimum geometry, which is substantially planar, the geometry at the S_2_/S_1_ CI is non-planar due to partial breakage of the π system. This molecular distortion causes rapid relaxation on to the S_1_ PES. A similar molecular distortion was determined at the S_1_/S_0_ CI, leading to rapid relaxation back the electronic ground state for gadusol. The computed MEPs find no barriers to either CI [87], providing evidence for the lack of fluorescence and high level of photostability observed for gadusol in the previous work by Arbeloa et al. [82] For gadusolate, Losantos et al. [87] determined that the first optically bright state was the S_1_ and had ^1^ππ* character. An easily accessible S_1_/S_0_ CI was located, with the main geometry distortions being an out-of-plane movement of the negatively charged oxygen on the chromophore and elongation of the C=C bond. Further to this, a small out-of-plane distortion of the C=O bond was also found at the S_1_/S_0_ CI. In summary, the geometry distortions at the CI points for both gadusol and gadusolate are similar [87]. The simpler deactivation pathway for gadusolate involving one CI may explain the lower photodecomposition quantum yield compared to gadusol [82,87].

**Figure 13:**
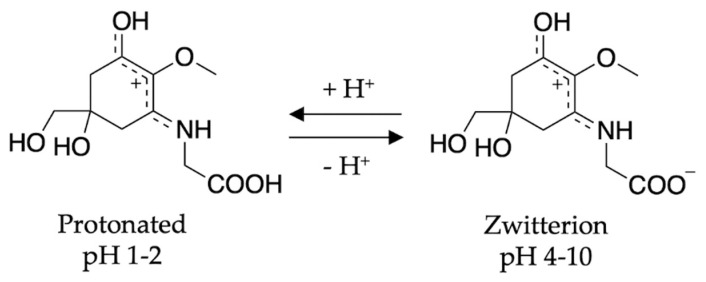
Structure of mycosporine-glycine at different pHs determined by Matsuyama et al. [129].

To conclude the mycosporine discussion, it is noted that additional studies on the mycosporines, mycosporine-glycine (see Figure 13 for structure) and mycosporine-glutaminol-glucoside, have also demonstrated high photostability, as only small decreases in their absorption spectra were observed after continuous UV irradiation [81,123]. Furthermore, a computational study on a model of mycosporine-glycine (glycine substituent at carbon three is exchanged for a NHCH_3_ group) by Matsuyama et al. [129] determined the species responsible for its UV absorption profileto be protonated on the oxygen at carbon one. In the same work, Matsuyama et al. [129] studied the effect of pH on mycosporine-glycine. The findings were that a small hypsochromic shift was observed for the absorption maximum at low pH (1–2) in comparison to pH 4 to 10 which remained unchanged. The shift at low pH was attributed to protonation of the carboxylate anion to give an overall charged species; see Figure 13 for clarity. From these findings, the authors concluded that the charge resonance through the chromophore, which is a result of the amino acid residue facilitating a zwitterionic structure (see Figure 13), is the reason for the low energy allowed transition observed in mycosporines, and is also responsible for their UV protective function. Matsuyama et al. [129] suggest that after photoexcitation, non-radiative decay of mycosporines likely occurs via the triplet state. However, Matsuyama et al.’s study does not map the MEPs or identify CIs to elucidate the photoprotection mechanism [129].

In summary, the additional substituents on the ring of mycosporines and gadusol/gadusolate compared to the simple mycosporine motifs may be the cause of the accessible S_1_/S_0_ CI. The accessible S_1_/S_0_ CI demonstrates how mycosporines act as UV filters for microorganisms in nature. Certainly, further investigation into structures with a cyclohexenone core are warranted, in particular, structures that more closely resemble mycosporines and progress away from the simple bottom-up units studied by Losantos et al. [89] and Woolley et al. [85]. Such investigations will provide greater insight into the photoprotective capabilities of mycosporines and nature’s choice to use them as UV filters. Furthermore, identifying substituents which improve the photoprotective properties of mycosporine motifs will, in turn, create a wealth of knowledge that can be applied to the development of nature-inspired UV filters for use in sunscreen formulations. It is noted here that the relaxation mechanism determined for mycosporines and mycosporine motifs differs from those found for the plant-inspired UV filters that were reviewed in Section 2.1. It is interesting that nature has adapted to synthesize these two different families of molecules, among a repertoire of different molecular approaches, to provide photoprotection for different life-forms.

#### 2.2.2. Mycosporine-Like Amino Acids

**Figure 14:**
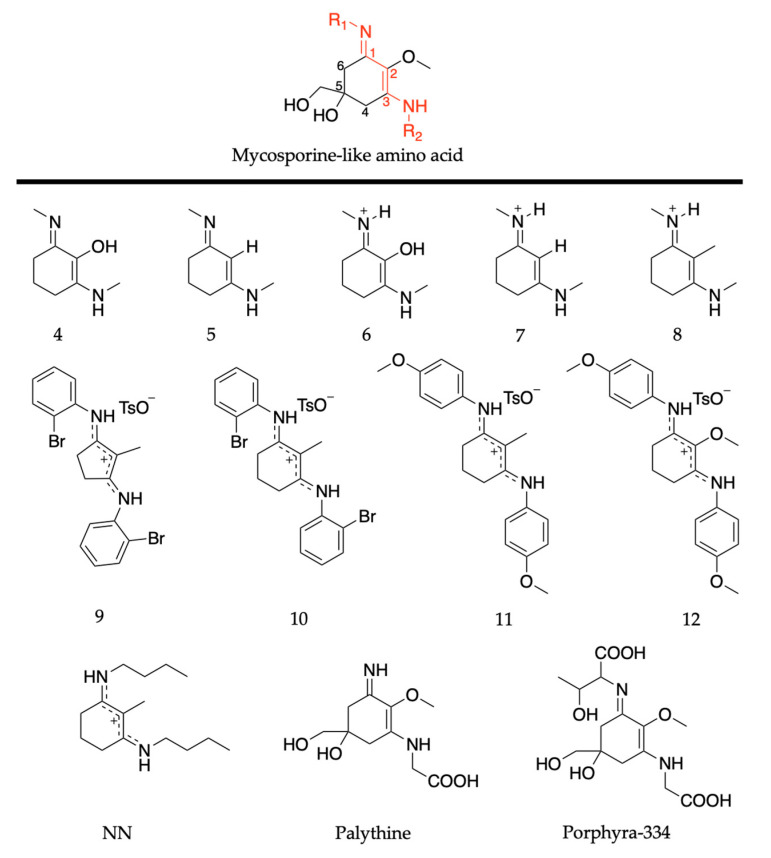
Structures of mycosporine-like amino acid (MAA) motifs studied by Losantos et al. [84,89] and Woolley et al. [85], and the natural MAAs studied by Sampedro [86], Koizumi et al. [88] and Hatakeyama et al. [90].

MAAs are composed of a cyclohexenimine core, with a methoxy group at carbon two, and hydroxy and hydroxymethyl substituents at carbon five; see Figure 14. Carbon one is substituted with an imino moiety (represented as R_1_ in Figure 14) and carbon three is substituted with an amino group (represented as R_2_ in Figure 14); this is what causes the variability among MAAs [60]. As with the mycosporine discussion in Section 2.2.1, this section of the review will begin with the MAA motif studies [85,89]. Only a brief overview is required as previous publications have reviewed these studies [55,83] (see Section 2.2.1 for more details). Losantos et al. [89] studied several molecules with a cyclohexenimine core, molecules 4–8 given in Figure 14, using the same CASPT2//CASSCF methodology [119,120] supra. Woolley et al. [85] used TEAS to experimentally investigate an MAA motif, (Z)-N-(3-(butylamino)-2-methylcyclohex-2-en-1-ylidene)butan-1-aminium 4-methylbenzenesulfonate (termed NN), also shown in Figure 14. All molecules are composed of a cyclohexenimine core, with different substituents on carbons one, two and three.

**Figure 15:**
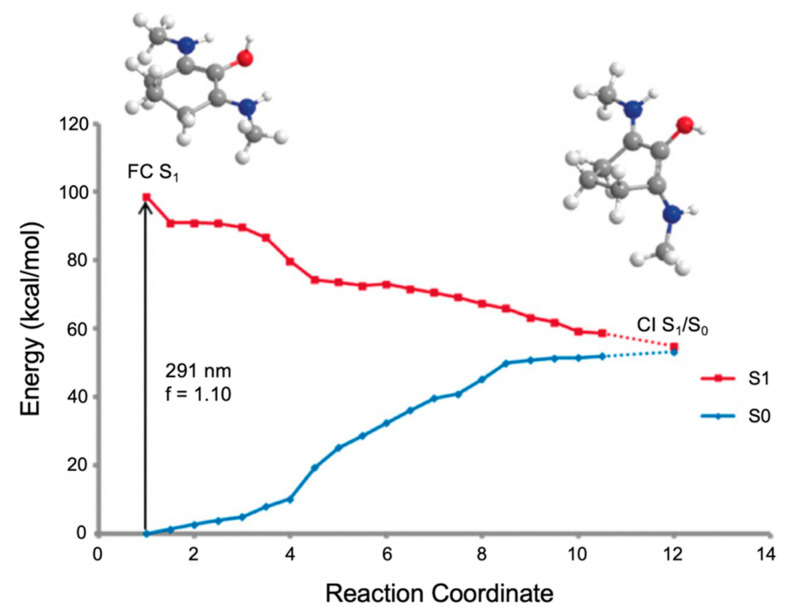
Computed MEP of molecule 6 by Losantos et al. [89], where f is the oscillator strength. Reproduced and adapted with permission from [89]. © 2017 Wiley-VCH Verlag GmbH & Co. KGaA, Weinheim.

The MEP for molecule 6 is displayed in Figure 15 and is representative of all the MAA motifs studied by Losantos et al. [89] For molecules 4–8, photoexcitation to the first singlet state, S_1_, occurs via a ^1^ππ* transition. The computed MEPs for molecules 4–8 all have a barrierless path towards a low energy S_1_/S_0_ CI. At the CI, the main geometric distortion is an out-of-plane movement of the substituents at carbons one, two and three. Therefore, the authors concluded that the decay process for molecules 4–8 will be ultrafast and non-radiative, which is ideal for a UV filter. Furthermore, the positive charge on molecules 6–8 red shifts the absorption band into the UV-B region, thus making them the more ideal cores out of the five computationally studied molecules (molecules 4–8, see Figure 14). Losantos et al. [89] chose molecules 6 and 8 for non-adiabatic molecular dynamics simulations to predict their S_1_ lifetimes. These were determined as ~190 and 240 fs for molecules 6 and 8, respectively, which is remarkably faster than the lifetimes known for some commercial UV filters [105,130,131]. Losantos et al.’s theoretical work guided the synthesis of 20 different molecules derived from molecules 6 and 8; molecules 9–12 in Figure 14 are a few of these [89]. Following this, steady-state measurements of these molecules were obtained within the same work. Very high levels of photostability and low fluorescence quantum yields were found for all synthesized molecules. Furthermore, the authors measured the solar protection factor and UV-A protection factor in real sunscreen formulations [89], prepared and measured following the industrial standard [132]. They found that when their synthesized MAA motifs were combined with commercial UV filters in a sunscreen formulation, an evident boost in both factors was observed compared to formulations containing only synthesized MAA motifs or commercial UV filters [89].

Building on Losantos et al.’s theoretical work [89], Woolley et al. [85] used TEAS to investigate NN experimentally. Methanol and acetonitrile were the solvents used and the appearance of both TAS did not display any noticeable differences. The authors determined that following photoexcitation to the S_1_ excited state, repopulation of the electronic ground state occurred on a timescale of ~ 500 fs. Furthermore, the NN TAS in both solvents displayed a weak absorption beyond the final time delay of Woolley et al.’s TEAS experiment, Δ*t* > 2.5 ns. This long-lived feature was attributed to any remaining photoexcited population either as a populated triplet state or as a photoproduct which is likely to have a low yield [85]. To summarize, the results for this study corroborate the theoretical work by Losantos et al. [89] that MAA motifs demonstrate promising photoprotective properties ideal for a UV filter.

**Figure 16:**
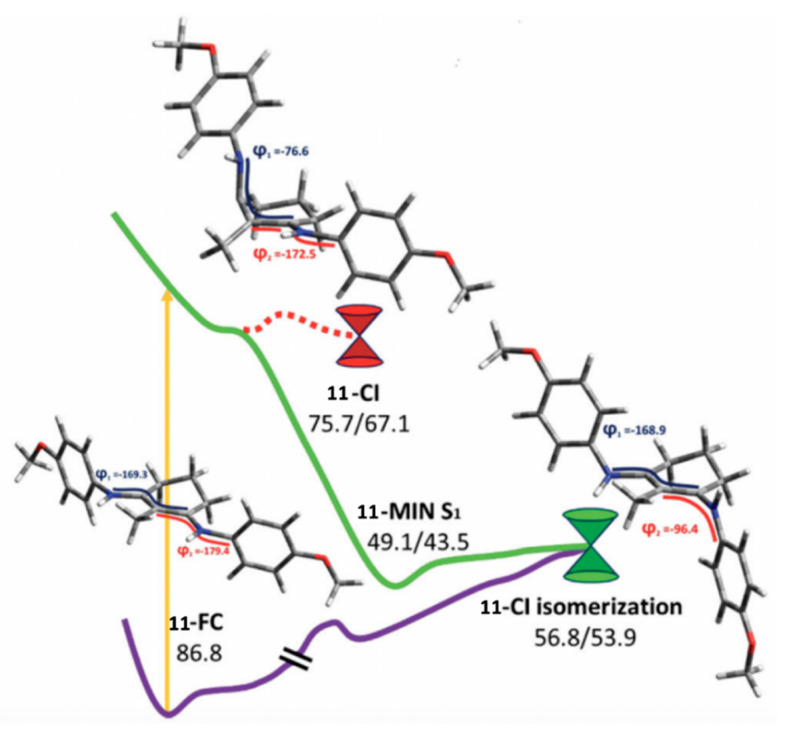
Critical points along the MEP for molecule 11 computed by Losantos et al. [84], where the energies are reported in kcal mol^-1^ with respect to the electronic ground-state minimum. Where two numbers are present, the first number corresponds to the S_1_ energy, and the second number corresponds to the S_0_ energy at the specified geometry. Reproduced from [84], with permission from the PCCP Owner Societies.

Following up on their earlier theoretical and experimental work [89], Losantos et al. [84] further investigated some of the UV filter candidates they previously synthesized using TEAS, fluorescence up-conversion and computational methods. The four studied molecules (9–12) can be found in Figure 14 [84]. CASPT2//CASSCF methodology [119,120] was implemented, similar to their previous study [89], to determine the deactivation pathway of molecule 11. Initial photoexcitation was found to be through an S_1_ ← S_0_ transition having clear ππ* character [84], similar to that found for the core components discussed above [89]. Two deactivation pathways were determined by Losantos et al. [84] and a PES diagram displaying these pathways is shown in Figure 16. The first pathway proceeds via a high-energy S_1_/S_0_ CI, with the main geometrical feature being that of an out-of-plane distortion of the substituents on the cyclohexenimine ring, like that found for the core structures in Losantos et al.’s previous study [89]. On further analysis, Losantos et al. [84] determined that this CI is not directly accessible from the Franck–Condon region. The second deactivation pathway follows a sharp descent from the Franck–Condon region to a minimum on the S_1_, whereby one of the imine moieties on molecule 11 is twisted out of the plane. The authors identified that isomerisation of one of the C=N bonds leads to a different S_1_/S_0_ CI which is slightly higher in energy compared to the S_1_ minimum, <8 kcal mol^−1^. Furthermore, no significant emission was observed at long timescales, indicating that molecule 11 can overcome the energy barrier and return to its electronic ground state. Analysis of the isomerisation CI concluded that isomerisation around one C=N is not efficient and an aborted isomerisation back to molecule 11′s original geometry was favoured [84].

**Figure 17:**
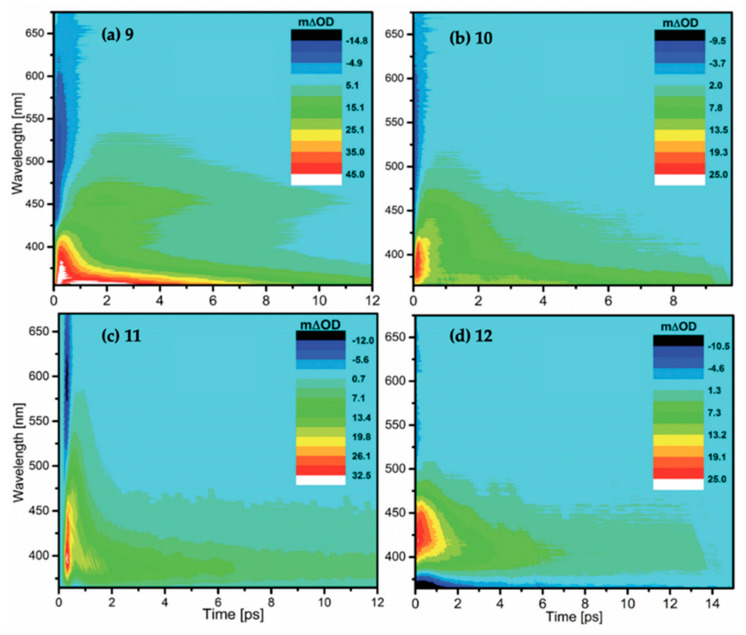
TAS displayed as false colour maps for molecules 9–12, respectively, in methanol photoexcited at (**a**) 306 nm, (**b**) 330 nm, (**c**) 341 nm and (**d**) 353 nm, studied by Losantos et al. [84]. Reproduced and adapted from [84], with permission from the PCCP Owner Societies.

The fluorescence up-conversion experiments performed by Losantos et al. [84] demonstrated fluorescence decay within hundreds of femtoseconds for molecules 9–12, thus indicating that the molecules relax to a non-fluorescent region after this time, which the authors conclude as being the electronic ground state of molecules 9–12. The TAS obtained by Losantos et al. [84] for molecules 9–12 in methanol are displayed in Figure 17 and the time constants extracted from a global fit function are presented in Table 4. The global fitting used by the authors was achieved using a convolution function given by;

(2) $$S\left(\lambda ,\;t\right)=\;\overset{\infty }{\underset{-\infty }{\int }}M\left(\lambda ,\;t-t^{\prime }\right)R\left(\lambda ,\;t^{\prime }\right)dt’$$

(3) M(t)= ∑i=1naie−tτi

where *M*(*t*) is a multi-exponential molecular response and *R*(*λ*,*t*) is the cross-correlation function. Implementation of this global analysis allows the extraction of a single set of time constants, *τ_i_*, for a collection of *a_i_*(*λ*) pre-exponential factors.

The TAS for molecules 9–12 displayed in Figure 17 all have a similar appearance. There is a negative feature (particularly weak in molecule 12) at early time delays extending to the red edge of the probe window which Losantos et al. [84] attributed to stimulated emission. In all TAS, there is a positive feature which covers a range of 450–550 nm (for molecules 9 and 10 the absorption extends to lower wavelengths). It is noted by the authors that the red edge of the absorption is shifted from zero, indicating that it is not formed directly from the initial photoexcitation. Losantos et al. [84] attribute this positive feature to a combination of ESA of the prepared excited state and a band corresponding to a second location post relaxation of the prepared excited state. Furthermore, a GSB can be observed for molecule 12 at ~350 nm and below.

Losantos et al. [84] extracted three time constants from their fits for each molecule—*τ*_0_, *τ*_1_ and *τ*_2_ for molecule 9 and *τ*_1_, *τ*_2_ and *τ*_3_ for molecules 10–12, see Table 4. The authors attribute *τ*_1_ to the evolution of the prepared excited state out of the Franck–Condon window towards the S_1_/S_0_ CI, whereby population to the vibrationally hot electronic ground state is achieved. The authors draw confidence with this assignment due to the good correlation between the *τ*_1_ lifetimes and the fluorescence up-conversion decay lifetimes. Losantos et al. [84] assigned *τ*_2_ and *τ*_3_ to vibrational cooling on the S_0_ state, with *τ*_3_ being vibrational cooling of colder states of the electronic ground state, which is corroborated by the blue shift of the absorption in the TAS (see Figure 17). For molecule 9, an early time constant, *τ*_0_, is required for the fit to model the early times of the initial relaxation of molecule 9 as it traverses along the S_1_ PES and evolves out of the Franck–Condon region. Furthermore, the fitting of molecule 9 did not extract a *τ*_3_, which Losantos et al. [84] explain as being due to the vibrationally hot electronic ground state being shifted to higher energies with respect to the other molecules.

A second interpretation of the time constants proposed by Losantos et al. [84] was that *τ*_1_ corresponds to evolution out of the Franck–Condon region on the S_1_ PES, *τ*_2_ corresponds to internal conversion from a relaxed S_1_ state to the vibrationally hot S_0_ state and *τ*_3_ corresponds to vibrational cooling on the electronic ground state. This second interpretation is proposed because as relaxation along the S_1_ PES occurs, fluorescence should occur at longer wavelengths which would result in fluorescence and stimulated emission extending out of the probe window in the TEAS and fluorescence up-conversion experiments. Therefore, it is difficult to determine which interpretation is correct. However, it is noted that this second time constant assignment does not alter the photophysical properties of the studied molecules other than them having a slightly longer S_1_ lifetime. Finally, Losantos et al. [84] did not observe any evidence for isomer formation and no long-lived feature is present in the TAS unlike Woolley et al.’s study on NN [85]. Losantos et al. [84] concluded their findings by stating that they believe the photoisomerisation reaction coordinate is the favoured deactivation pathway for these phenyl-substituted MAA motifs. Their experimental data were unable to confirm this; however, this is likely due to the aborted photoisomerisation leading to recovery of the original electronic ground state of molecules 9–12 predicted by the computational calculations in their study [84]. In conclusion, the results from Losantos et al.’s studies are highly promising for sunscreen applications, as molecules 9–12 display efficient repopulation of the electronic ground state non-radiatively on ultrafast timescales [84], low fluorescence and high photostability [89]. In this work, the authors explored a new family of MAA motifs containing phenyl substituents [84], and determined that they follow a different relaxation mechanism upon photoexcitation compared to the previously proposed ring distortion from their earlier work on simple MAA motifs [89]. Taken together, this highlights that the substituent on the core structure of MAAs plays an important role in determining the relaxation mechanism that is observed. However, in the above examples, both mechanisms are highly effective for the role of a UV filter in sunscreen formulation.

Within the literature, there have been computational studies on the natural MAAs palythine [86] and porphyra-334 [88,90]; see Figure 14 for their respective structures. The MEPs of these MAAs are not reproduced here due to the similarity between those that have been presented throughout Section 2.2 of this review. Beginning with palythine, the same CASPT2//CASSCF methodology [119,120] described above was implemented by Sampedro [86] and this work has been reviewed previously [62]. Both a neutral and protonated form of palythine was studied, and it was confirmed that the protonated form is the species that contributes the most towards the photoprotective capabilities of palythine. However, dependent on pH, it is likely that both species are present in solution, hence the author’s choice to evaluate both MEPs. In the case of the neutral form, two different absorption bands were calculated: S_1_ ← S_0_ having nπ* character, and S_2_ ← S_0_ having ππ* character and a greater oscillator strength than the former absorption band. After photoexcitation to the S_2_, rapid deactivation via two energetically accessible CIs, S_2_/S_1_ and S_1_/S_0_, is reached by small geometrical changes resulting in repopulation of the vibrationally hot electronic ground state. On relaxation along the S_0_ PES, the ground-state minimum of palythine is reached along with a slightly higher in energy (1.5 kcal mol^-1^) conformer. Upon direct photoexcitation to the S_1_, Sampedro [86] reported that the same S_1_/S_0_ CI is easily accessible and leads to the formation of the same conformer supra.

For the protonated form of palythine, Losantos et al. [86] calculated only one absorption band—a S_1_ ← S_0_ transition with ππ* character. Upon photoexcitation to the S_1_ state, a geometric distortion results in palythine reaching an accessible S_1_/S_0_ CI. This leads to efficient repopulation of the electronic ground state, which relaxes to both the original protonated form and its slightly higher energy conformer as was found for the neutral form of palythine. Analysis of the S_1_/S_0_ CI determined that the geometric changes are out-of-plane movements of the imine moiety, while the adjacent carbons approach each other, and the alkene within the chromophore remains almost planar [86], similar to that reported for the core structures of MAAs by Losantos et al. [89].

In conclusion, both the protonated and neutral forms of palythine dissipate their absorbed energy rapidly into heat. However, due to the strong absorption closer to the reported experimental value, Sampedro [86] concluded that the protonated form most likely undertakes the UV protective role. In addition, photoacoustic calorimetry performed by Conde et al. [77] confirmed that palythine relaxes mostly through non-radiative decay pathways and, further to this, has a photodegradation quantum yield of 1.2 × 10^−5^, corroborating the conclusions made by Sampedro [86]. This evidence for non-radiative decay resulting in efficient repopulation of the electronic ground state supports MAAs for their function as UV filters. As mentioned in Section 2.2.1 of this review, details on the experimental setup of photoacoustic calorimetry and photoacoustic calorimetry on MAAs specifically has been reviewed in previous publications [62,83,125,126,127].

Two complementary computational studies have been conducted on porphyra-334 to unravel its photoprotective mechanism [88,90]. The work by Koizumi et al. [88] used first-principles molecular dynamics [133] on hydrated porphyra-334 to investigate the mechanism that photoexcited porphyra-334 undergoes to dissipate excess energy to surrounding water. The findings were that energy transfer occurred mainly through the activation of hydrogen-bond stretching modes among water molecules. Only the hydrogen-bond network of the solvent was disrupted during the simulation, and the first hydration shell of porphyra-334 remained unchanged throughout. The tightness of the first hydration shell hydrogen bonds to porphyra-334 indicates how efficient energy transfer can occur without disruption to chemical bonds in both the solute and the solvent [88].

The later work by Hatakeyama et al. [90] investigated the electronic states of porphyra-334 using quantum chemical simulations, in order to complete the understanding of the relaxation mechanism it undergoes to return to its electronic ground state. In this work, the zwitterionic and less polar neutral forms of porphyra-334 were studied. After initial excitation to the S_1_, relaxation along the S_1_ PES leads to a S_1_/S_0_ CI with non-planar geometry (primarily at the CN groups at carbons one and three), this is in contrast to the planar geometry found for the optimized S_0_ structure. This deformation results in rapid deactivation of the electronic excited state [90], similar to that reported for protonated palythine [86] and the core components of MAAs [89]. Hatakeyama et al. [90] associate this geometry change with a π electron shift from the cyclohexene ring towards the protonated CN group of the glycine or threonine arm, which triggers the sp^3^ hybridisation of the CN groups in the S_1_ state. As the photoprotective mechanism is similar to those already reported in Section 2.2, no further details are given here. Porphyra-334 has also been studied experimentally by Conde et al. [75], who determined the fluorescence quantum yield to be 0.0016, the triplet formation quantum yield to be <0.05 and the photodecomposition quantum yield to be 2–4 × 10^−4^. These very low quantum yields lead to Conde et al. [75] concluding that porphyra-334 undergoes a rapid internal conversion from its electronic excited state to its electronic ground state, which is corroborated by the theoretical work [88,90]. Later work using photoacoustic calorimetry by Conde et al. [76] quantitatively determined that between 96 and 98% of the absorbed energy upon photoexcitation is rapidly released to the surroundings as heat for porphyra-334. The efficient repopulation of the electronic ground state, low fluorescence quantum yields and low triplet formation quantum yields for porphyra-334 highlights the promising characteristics that are required for a UV filter in sunscreen formulation. Such properties lower the probability for competing side reactions, which may result in harmful photoproduct or radical formation. 

**Figure 18:**
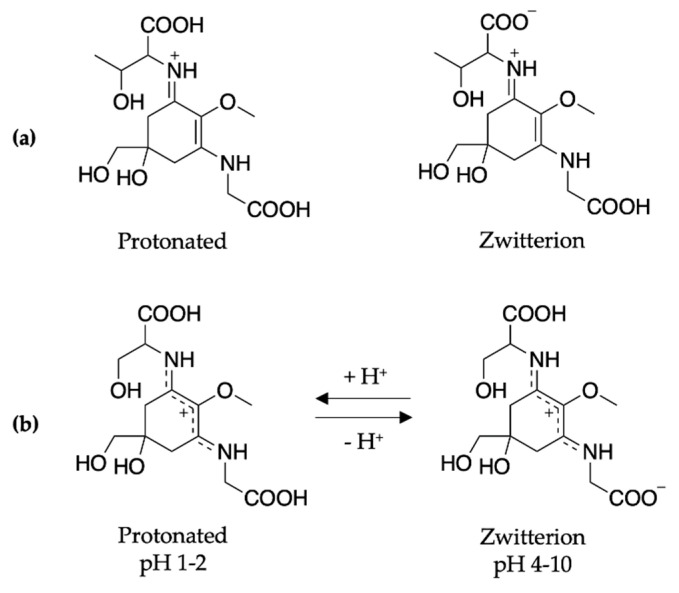
(**a**) Structure of porphyra-334 elucidated by Klisch et al. [134], and (**b**) structure of shinorine at different pHs determined by Matsuyama et al. [129].

Moreover, a previous publication by Klisch et al. [134] elucidated the structure of porphyra-334 to most likely be protonated on the imine nitrogen on carbon one (either as a zwitterion or as the protonated form; see Figure 18a). This was concluded by comparing density functional theory (DFT) chemical shift predictions to several experimental nuclear magnetic resonance (NMR) spectra. Whilst Sampedro [86] determined that protonated palythine is responsible for the UV-absorbing properties compared to its neutral form, and Hatakeyama et al. [90] determined that the zwitterionic form of porphyra-334 was responsible for the UV-absorbing properties, neither study performed a comparison between the protonated form and the zwitterionic form. A study on shinorine by Matsuyama et al. [129] performed such calculations, and took experimental data over a broad pH range (1–10). The findings were the same as those discussed in Section 2.2.1 for mycosporine-glycine; over the pH range 4–10, the maximum absorbance did not change, but, at pH 1 to 2, a small hypsochromic shift was observed. Quantum chemical calculations revealed that protonation on the chromophore, which facilitates charge resonance, permits the low energy allowed transition observed in MAAs and is responsible for their UV protective function. At pH 4–10, the overall species in neutral, and at pH 1–2, further protonation of the carboxylate anion generates an overall charged species; see Figure 18b for clarity. Additionally, they found shinorine to have a low fluorescence quantum yield (2 × 10^−4^), similar to the fluorescence quantum yield previously reported by Conde et al. [76] ((1.6 ± 0.2) × 10^−4^). This lead to the authors’ conclusion that the dominant relaxation pathway is non-radiative, possibly via the triplet state. Again, it is added here that Matsuyama et al.’s study did not directly examine the MEPs or CIs to determine the relaxation pathway [129]. In addition, Conde et al. [76] determined that the triplet formation quantum yield for shinorine was <0.05, and that between 96 and 98% of shinorine rapidly dissipates absorbed UVR as heat to its surroundings. Taken together, these findings highlight the photoprotective capabilities of MAAs; rapid non-radiative decay dominates, lowering the chance for competing reactive pathways that may result in harmful photoproducts. Furthermore, Klisch et al. [134] found that the computed proton affinity of neutral porphyra-334 was high and, as a result, it behaves as a ‘proton sponge’. The authors attribute this to porphyra-334′s ability to delocalize the positive charge throughout the chromophore. This is further corroborated by Zhang et al.’s study, which found porphyra-334 to be stable below pH 12 but decompose at pH 12 and above [135]. Such knowledge may have important implications for the commercial industry during formulation production in choosing ingredients and pHs to work at.

Recently, Orallo et al. [136,137] have performed studies on the MAAs shinorine, porphyra-334 and shinorine dimethyl ester in micellar solutions to elucidate the photochemistry and photophysics in a closer-to-real sunscreen formulation environment. They found that the fluorescence quantum yield and lifetime were increased in micelle solutions compared to aqueous solutions; however, the main deactivation process remained via non-radiative decay. Additionally, the photodecomposition quantum yields decreased when the MAAs were in micelle solutions. The authors attributed both properties to the electrostatic interactions between MAAs and micelles which hinder molecular movements. Overall, Orallo et al. [136,137] demonstrated that MAAs in micellar solutions which mimic a closer-to-real environment, with regards to sunscreen formulation, still perform as efficient UV filters which is promising for commercial applications. However, the electrostatic interactions which influence the decay mechanisms must be taken into account.

In summary, the photoprotective mechanism for MAAs determined so far, taking palythine [86] and porphyra-334 [90] as examples, are very similar to the photoprotective mechanism identified for the basic core components of MAAs [89]. This photoprotective mechanism occurs through the main geometric deformation of an out-of-plane distortion of the substituents on the ring. However, this is not the case when phenyl rings are substituted on to the core structure and instead deactivation favours the aborted isomerisation pathway [84]; this is similar to the decay mechanism observed for plant-inspired UV filters (see Section 2.1), the difference being that evidence for the generation of both isomers was present for those molecules. Further work is certainly warranted on the effect of the substituents on the ring and the favoured deactivation pathway as a result. Nevertheless, all work conducted on MAAs and MAA motifs have demonstrated desirable properties of UV filters and this deeper understanding of the photoprotective mechanisms could help guide the future design on UV filters.

## 3. Future Direction for Nature-Inspired Ultraviolet Filters

In terms of the future direction for nature-inspired UV filters, the work by Horbury et al. [115] carried out on DES (Section 2.1.3) and Orallo et al. [136,137] carried out on MAAs (Section 2.2.2) serve as pertinent examples of the need to study the photodynamics of potential UV filters in a closer-to-real environment. It is important that such work is continued across UV filter research in order to determine any differences between the photodynamics of the targeted molecule in solution and on a skin mimic. The closer to real life the system studied is, the more pertinent the results are to real-world application. Furthermore, an improved knowledge of how UV filters behave on a skin mimic will ultimately aid future design of UV filters.

To date, the study of nature-inspired UV filters utilising both theory and TEAS to unravel their dynamical properties following UV absorption has received considerable attention. Introducing the vibrational analogue of TEAS, transient vibrational absorption spectroscopy (TVAS) to study nature-inspired UV filters could aid their molecular design, given that TVAS can detect molecular structural changes, such as chemical bond formation or breakage. Such processes cannot be confirmed by probing the electronic state alone using TEAS. At present, there is only one example of the use of TVAS for sunscreen applications—a study on oxybenzone by Baker et al. [105]. In addition to the ability TVAS has to detect molecular structural changes, it is also possible to extract GSB recovery quantum yields [138,139,140,141], something that is again difficult to achieve in a TEAS experiment. For example, in Baker et al.’s study, photoproduct formation quantum yields were reported and this was achieved through analysis of the incomplete GSB recovery from their TVAS spectra [105]. In combination with computational calculations, TVAS could be a very powerful technique for aiding the analysis of the photodynamical processes occurring in nature-inspired UV filters, and more broadly speaking, all UV filters.

Additionally, the study of nature-inspired UV filters could benefit from the application of two-dimensional electronic spectroscopy (2DES) and its vibrational analogue, two-dimensional vibrational spectroscopy (2DVS). The 2DES is an ultrafast laser spectroscopy used to monitor the energy and spatial landscape of a system in an electronically excited state following interaction with a sequence of three laser pulses. It is particularly useful in systems with multiple interacting components for tracking excited-state energy transfer processes. Likewise, it allows one to extract the species responsible for an absorption or emission signal observed in the excited state [142,143]. This information is often difficult to observe by probing the electronic state using TEAS. On the other hand, 2DVS provides information relating to the system under investigation in the electronic ground state, following photoexcitation. While 2DES and 2DVS have found applications in exploring the photochemistry of biological and material samples [144,145,146], there is currently no literature reporting their use in UV filter applications. In combination with TEAS, TVAS and computational methods, 2DES and 2DVS could provide complementary information aiding the understanding of UV filter photodynamics and their molecular design. 

## 4. Conclusions

In conclusion, this review has outlined the developments being made in investigating nature-inspired UV filters. Two distinctive directions have been highlighted—plant-inspired and microbial-inspired UV filters. To summarize, the UV spectra and photostability studies on nature-inspired UV filters reported within the literature have revealed that they absorb UV strongly in the region of UV-B and UV-A and are photostable. This is promising, given the sparsity of photostable commercial UV-A filters. Further, the photoprotective mechanisms for plant-inspired UV filters presented in Section 2.1 largely involve efficient and non-radiative relaxation to dissipate the absorbed energy via a *trans–cis* photoisomerisation. This relaxation pathway often results in the formation of the isomer as a long-lived photoproduct. As discussed, recent work by Horbury et al. [115], reviewed in Section 2.1.3, has highlighted that appropriate structural modifications could prevent the formation of the isomer photoproduct. In the case of most microbial-inspired UV filters presented in Section 2.2, the proposed photoprotective mechanism involves an out-of-plane geometric distortion of the substituents on the ring. However, when MAA motifs are substituted with a phenyl ring, a second photoprotective mechanism becomes available; this being an aborted photoisomerisation pathway which was determined through computational calculations and the absence of isomer formation. This differs from the results observed for plant-inspired UV filters, as isomer formation was evident for all except the symmetrically functionalized sinapate ester, of which the *cis* and *trans* isomers are the same. Both families of nature-inspired UV filters warrant further work to improve the knowledge of the photoprotective mechanisms understood thus far. In particular, substituent effect is an area to be further investigated in the hope of enhancing future UV filter design. Furthermore, this review has touched on work that has mimicked a close-to-real environment and highlighted how important such experiments are towards understanding the photoprotective mechanisms for the application of UV filters in commercial sunscreens. Finally, promising UV filter candidates have been identified throughout this review and, with future work, it is possible that next-generation UV filters could be nature-inspired. With this in mind, nature-inspired UV filters could tackle some of the drawbacks currently faced centred around human and ecotoxic concerns of current UV filters.

## Figures and Tables

**Table 1:** Summary of lifetimes of dynamical processes of SM and SA. Reproduced and adapted with permission from [104], licensed under CC-BY. © 2015 American Chemical Society.

	*τ_n_*	Dioxane	Acetonitrile	Methanol
**SA**	*τ*_1_/fs	93 ± 17	52 ± 5	572 ± 87
	*τ*_2_/ps	0.90 ± 0.19	0.57 ± 0.04	3.79 ± 0.72
	*τ*_3_/ps	12.2 ± 1.1	17.0 ± 0.66	25.5 ± 1.6
**SM**	*τ*_1_/fs	119 ± 28	51 ± 4	619 ± 101
	*τ*_2_/ps	1.62 ± 0.15	0.63 ± 0.04	4.81 ± 0.77
	*τ*_3_/ps	22.4 ± 1.9	27.3 ± 0.77	33.5 ± 1.7

**Table 2:** Summary of the lifetimes of dynamical processes of *cis*-ES and *trans*-ES. Reproduced and adapted with permission from [114], licensed under CC BY 3.0. Published by the Royal Society of Chemistry.

	*τ*_ivr_ (fs)	*τ*_iso_ (ps)	*τ*_pp_ (ns)
*cis*-ES	330 ± 40	5.05 ± 0.06	>>2
*trans*-ES	290 ± 40	4.60 ± 0.04	>>2

**Table 3:** Summary of the rate constants (*k_n_*) resulting from sequential global fit of the TAS of DES in VC/AB, AB, ethanol and cyclohexene. Reproduced and adapted with permission from [115], licensed under CC BY 4.0. Published by Springer Nature.

	*k*_1_/s^−1^ (×10^13^)	*k*_2_/s^−1^ (×10^12^)	*k*_3_/s^−1^ (×10^11^)	*k*_4_/s^−1^ (×10^10^)	*k*_5_/s^−1^ (×10^8^)
VC/AB	0.7 ± 0.2	3.0 ± 0.3	4.24 ± 0.07	1.02 ± 0.06	<<5
AB	2.5 ± 2.5	2.1 ± 0.2	5.3 ± 0.1	2.6 ± 0.1	<<5
Ethanol	1.1 ± 0.5	11 ± 5	12.6 ± 0.6	15.5 ± 0.1	<<5
Cyclohexane	0.7 ± 0.2	n/a	16 ± 1	6.3 ± 0.2	n/a

**Table 4:** Summary of time constants resulting from the global fit of the TAS of molecules 9–12 in methanol studied by Losantos et al. [84]. Reproduced and adapted from [84], with permission from the PCCP Owner Societies.

	*τ*_0_	*τ*_1_	*τ*_2_	*τ*_3_
9	70 ± 21 fs	415 ± 43 fs	5 ± 0.22 ps	-
10	-	320 ± 110 fs	1.7 ± 0.48 ps	9.7 ± 1.04 ps
11	-	206 ± 65 fs	397 ± 190 fs	6 ± 0.4 ps
12	-	872 ± 201 fs	1.8 ± 0.15 ps	10.2 ± 0.22 ps
